# Biodegradation kinetics of organic micropollutants in biofilters for advanced wastewater treatment – Impact of operational conditions and biomass origin on removal

**DOI:** 10.1016/j.wroa.2024.100235

**Published:** 2024-07-08

**Authors:** Tobias Kaiser, Thomas Fundneider, Susanne Lackner

**Affiliations:** a Technical University of Darmstadt, Institute IWAR, Chair of Water and Environmental Biotechnology, Franziska-Braun-Straße 7, 64287 Darmstadt, Germany; b Mecana AG, Industriestrasse 39, 8864 Reichenburg, Switzerland

**Keywords:** Biofilm, Model, AQUASIM, Porous media, Pseudo first-order kinetics

## Abstract

Biofiltration processes are often part of advanced wastewater treatment (aWWT) technologies for the removal of organic micropollutants (OMP) from conventional wastewater treatment plant (WWTP) effluents. Although biological effects are not always the main focus of these technologies (e.g. filtration through granular activated carbon), they have been shown to contribute significantly to total OMP removal. While OMP biodegradation kinetics in conventional biological wastewater treatment are well researched, no systematic comparison to biomass from aWWT is available. This biomass faces different growth conditions and higher OMP concentrations relative to the background organic matter. Adaptation to these conditions could be possible and could lead to faster OMP biodegradation kinetics, which would show in a larger pseudo first-order biodegradation kinetic constant k_biol_. In this work, k_biol_ values for biomass obtained from aWWT biofilters were determined by evaluating OMP removals measured in lab-scale biofilters using a mechanistic model of the experimental setup. A comparison to k_biol_ values from literature for conventional wastewater treatment (with nutrient removal) revealed similar OMP biodegradation kinetics without any advantages of biomass from aWWT. A conceptual evaluation of influencing factors on OMP removal in biofilters showed that operational parameters (such as the biomass concentration or the empty bed contact time) and the affinity of OMPs to adsorb on biomass have a significant additional effect on biological OMP removal. Therefore, k_biol_ values alone are not sufficient to estimate biological OMP removal in biofilters and further information about the system is required.

## Highlights

• Micropollutant biodegradation in advanced wastewater treatment was investigated.
• Kinetic constants were determined using a mechanistic biofilter model.
• No differences were found compared to kinetics for biomass from secondary treatment.
• Kinetic constants alone do not allow conclusions on expected biological removal.

## Graphical abstract

**Figure:**
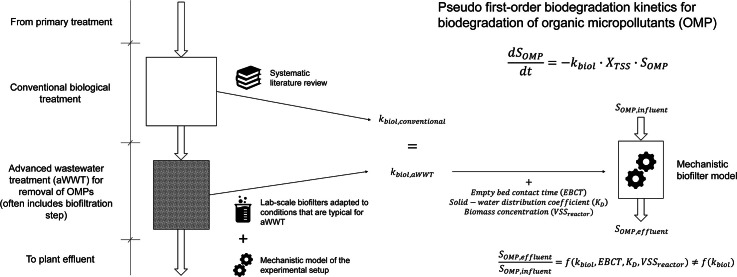

## Nomenclature

**Symbol**
A_biofilter_: Cross-sectional area of the biofilter, L²
A_F_: External surface area of carrier material, L²
B: OMP concentration sorbed on biomass, M/L³
EBCT: Empty bed contact time, T
f_diff_: Molecular diffusivity reduction constant for diffusion in biofilms, -
H: Dimensionless Henry's law constant, -
h_biofilter_: Height of the biofilter, L
k_biol_: Pseudo first-order biodegradation kinetics constant, L³/(M·T)
K_D_: Solid-water distribution coefficient, L³/M
k_sor_: Sorption rate constant, L³/(M·T)
L_BF_: Total biofilm thickness, L
L_BF,i_: Thickness of biofilm layer i, L
L_MTBL_: Mass transfer boundary layer thickness, L
m_TSS_: Absolute TSS mass in the reactor, M
m_VSS_: Absolute VSS mass in the reactor, M
Q: Flow rate, L³/T
q_ex_: Exchange coefficient in Aquasim for the saturated soil column compartment, L²/T
q_G_: Air applied per volume of reactor and time, L³/(L³·d)
r_g_: Mean grain radius of filter bed material, L
S_0_: Initial concentration of OMPs in the buffer tank (recirculation setup) or OMP influent concentration (conceptual setup), M/L³
S_0,biofilter_: Initial OMP concentration in the biofilter, M/L³
S: OMP concentration, M/L³
S_biofilter_: OMP concentration in the biofilter, M/L³
S_biofilter,eff_: Effluent OMP concentration from the biofilter, M/L³
S_biofilter,in_: Influent OMP concentration into the biofilter, M/L³
t: Time, T
V_BF_: Volume of the biofilm, L³
V_biofilter_: Volume of the biofilter, L³
V_buffer_: Volume of the buffer tank, L³
V_displacement_: Displacement volume of the filter bed material, L³
X_TSS_: Total suspended solids concentration, M/L³
VSS_reactor_: VSS concentration in the biofilter with regard to the reactor volume, M/L³
x: Spatial variable in direction of flow through the biofilter, L
Θ_Bulk_: Bulk volume fraction of total reactor volume, -
Θ_BF,i_: Volume fraction of biofilm layer i, -

## 1 Introduction

Advanced wastewater treatment (aWWT) is a general term for treatment stages downstream of the secondary clarification on municipal wastewater treatment plants (WWTP) (Tchobanoglous et al., 2014). aWWT processes that aim at removing organic micropollutants (OMP) are usually based on adsorption and/or oxidation processes. Powdered activated carbon adsorption (Boehler et al., 2012), granular activated carbon (GAC) filtration (Fundneider et al., 2021a; Kessler et al., 2022) or ozonation with subsequent sand filtration (Hollender et al., 2009) are typical examples. Both GAC and sand filters become biologically active as biomass adheres to and grows on the filter bed material (Çeçen and Aktas, 2011; Sbardella et al., 2018; Velten et al., 2011). It has been shown that these biological processes also contribute to OMP removal (Fundneider et al., 2021b; Hollender et al., 2009; Reungoat et al., 2011; Sbardella et al., 2018).

The biodegradation of OMPs is generally considered to be a co-metabolic process, meaning that it does not contribute to microbial growth (Arp et al., 2001). In the literature, the mathematical description of this process often follows simple pseudo first-order biodegradation kinetics as illustrated by Eq. (1). S is the OMP concentration, k_biol_ is the pseudo first-order biodegradation kinetic constant and X_TSS_ the concentration of the total suspended solids (TSS). The parameter k_biol_ provides a direct measure of OMP biodegradability and, in theory, enables comparability across different studies and systems and even predictions of expected OMP removal. This kinetic expression has already been used for both suspended and attached growth processes (Falås et al., 2013; Joss et al., 2006; Wolff et al., 2021). There are also more comprehensive kinetic approaches to predict biodegradation of OMPs (see Plósz et al. (2012), Sathyamoorthy et al. (2013), Fernandez-Fontaina et al. (2014)), but their application requires a deeper understanding of the system and sufficient data, which is often lacking.

(1) $$\frac{dS}{dt}=-k_{biol}\cdot X_{TSS}\cdot S$$

In contrast to the conventional biological treatment stage of a WWTP (defined in this work as processes that remove biodegradable organic matter and nutrients using microorganisms), downstream biofilters for aWWT are typically characterized by an influent with low concentrations of organic matter and nutrients. Moreover, the proportion of OMPs in all organic matter actually increases during conventional biological treatment because biological removal of most OMPs is moderate to poor in these treatment stages (Joss et al., 2006). Biomass can adapt to these environmental conditions, which might result in an increase of the k_biol_ values. The findings that biofilms show larger k_biol_ values than suspended biomass for some OMPs (Falås et al., 2013; Wolff et al., 2021) and that the microbial community in biofilters differs from the one in their influent (Zhang et al., 2018) supports the possibility that OMP biodegradation kinetics of biomass in biofilters for aWWT may indeed be more favorable compared to biomass from conventional biological wastewater treatment. However, k_biol_ values specifically determined for biofilters in aWWT (in the form specified by Eq. (1)) are not available in the literature.

This work aims at closing this knowledge gap. For this purpose, the OMP effluent concentrations of two lab-scale biofilters were considered. Each biofilter was inoculated with biomass from the backwash water of a full-scale GAC filter for aWWT. The GAC filters were operated on two different WWTPs and one of them had an upstream membrane filtration unit for suspended solids removal. Both lab-scale biofilters were operated in sequential batch mode with recirculation of membrane filtrated secondary effluent. Data was collected for two different batch runs after an initial operating period of 20 weeks. These two sampling campaigns included measurements of OMP effluent concentrations at specified time intervals. A simple mechanistic model of the experimental setup was used to estimate values of k_biol_ by fitting simulated OMP effluent concentrations to the corresponding measured datasets. The model considered biodegradation, sorption to biomass and volatilization of OMPs. The obtained k_biol_ values were eventually compared to the available literature ranges for each OMP, which were all based on OMP removal during conventional biological wastewater treatment. Finally, a conceptual evaluation of multiple influencing factors on OMP removal in biofilters used for aWWT was conducted by using a modified version of the mechanistic model and the obtained k_biol_ values. The following research questions were addressed in this work:

1. Does biomass from biofilters for aWWT systematically differ from biomass from conventional biological wastewater treatment in terms of OMP biodegradation kinetics?
2. Are k_biol_ values sufficient to roughly estimate the expected biological removal in such biofilters?

## 2 Results and discussion

### 2.1 Lab-scale biofilter performance and operating conditions

Four datasets resulted from the two sampling campaigns (one dataset for sampling campaign 1 and 2 for each biofilter 1 and 2). They consisted of initial OMP concentrations in the feed (at time t = 0 of the respective batch run) and the subsequent concentrations in the biofilter effluent over time. Additionally, the concentrations of the parameters ammonia nitrogen (NH_4_-N), phosphate phosphorus (PO_4_-P), dissolved organic carbon (DOC) and dissolved oxygen (DO) as well as values for the UV absorbance at 254 nm (UV_254nm_) are provided in SI Table 14 to SI Table 16. NH_4_-N concentrations were below the limit of quantification (LOQ, = 0.015 mg NH_4_-N/L) for both sampling campaigns, so the systems were probably limited in NH_4_-N. DO concentrations were always > 5 mg/L, indicating that aerobic conditions prevailed. Initial and effluent concentrations of 53 OMPs (known metabolites were excluded from the list of all measured OMPs) for each biofilter and sampling campaign are presented in SI Table 1 and SI Table 20 to SI Table 24. OMP removal took place in all the reactors and the extent of OMP removal was influenced by both OMP type and biomass quantity (see SI Table 13). For further model-based evaluation, only OMP concentrations above the LOQ were used and only OMPs for which sufficient data for model application was available were considered (24 OMPs, see SI Table 1).

### 2.2 OMP biodegradation kinetics in biofilters used for advanced wastewater treatment

**Fig. 1:**
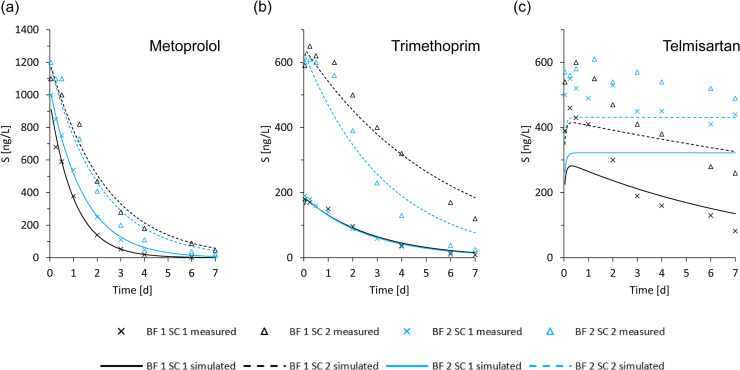
Graphical visualization of measured and simulated biofilter effluent concentrations, plotted over the batch operation time. Selection of 3 OMPs for the exemplary representation of the model accuracy: (a) metoprolol, (b) trimethoprim and (c) telmisartan. See SI Figure 5 to SI Figure 7 for other OMPs for which the model-based determination of k_biol_ was performed. BF stands for biofilter and SC for sampling campaign.

The reliability of the individual estimates for k_biol_ was derived from the deviation between measured and predicted OMP effluent concentrations (expressed as root mean square error (RMSE)). Estimates for k_biol_ were assumed to be more reliable with lower RMSE values of the corresponding model prediction. Fig. 1(a) and (b) exemplary shows two examples of a good agreement between measured and predicted OMP effluent concentrations (OMPs metoprolol and trimethoprim). Most OMPs showed a similarly good agreement (see SI Figure 5 to SI Figure 7) with a ratio of RMSE to initial batch concentration S_0_ for the fourfold k_biol_ estimation between 5 and 10% (see SI Figure 4). Therefore, the determined k_biol_ values were classified as reliable if the RMSE/S_0_ ratio was < 20%. Only telmisartan did not meet this criterion for each of the fourfold k_biol_ estimates, see also Fig. 1(c). Consequently, it was excluded from further analysis.

**Fig. 2:**
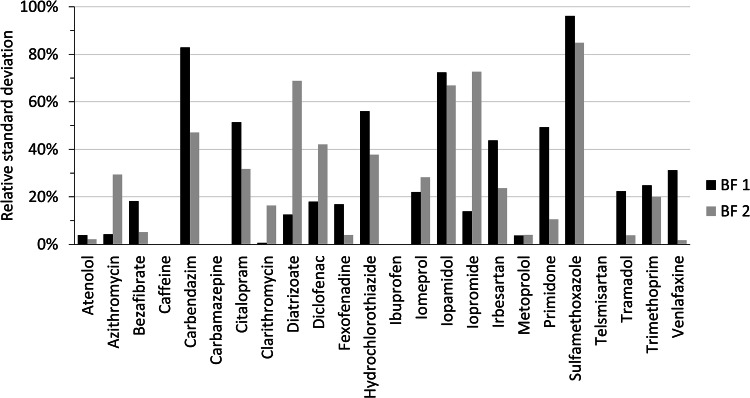
Relative standard deviation (RSD) of twofold k_biol_ determination of lab-scale biofilters BF 1 and BF 2 (one value of k_biol_ per sampling campaign and biofilter, respectively). OMPs without data were either excluded from further analysis (telmisartan) or only one dataset per biofilter was available (caffeine and iomeprol). Carbamazepine actually showed a RSD of 0%.

Individual estimates of k_biol_ for each OMP and biofilter (i.e. usually two values resulting from the two sampling campaigns) showed a certain degree of variation. For both biofilters, 50% of the considered OMPs had a relative standard deviation (RSD) of the two k_biol_ values that was ≤ 20%, see Fig. 2. For 80% of the considered OMPs, the RSD was still ≤ 50%. This can be seen as a fairly constant outcome overall, as a certain degree of variability in data resulting from biodegradation experiments was to be expected. For instance, differences in biomass composition between the different batch runs of the same biofilter cannot be ruled out. Large differences in RSD (≥ 30%) between the two biofilters occurred for the OMPs carbendazim, diatrizoate, iopromide and primidone. Interestingly, in general none of the two biofilters provided more consistent k_biol_ values (i.e. lower RSD) than the other one.

**Fig. 3:**
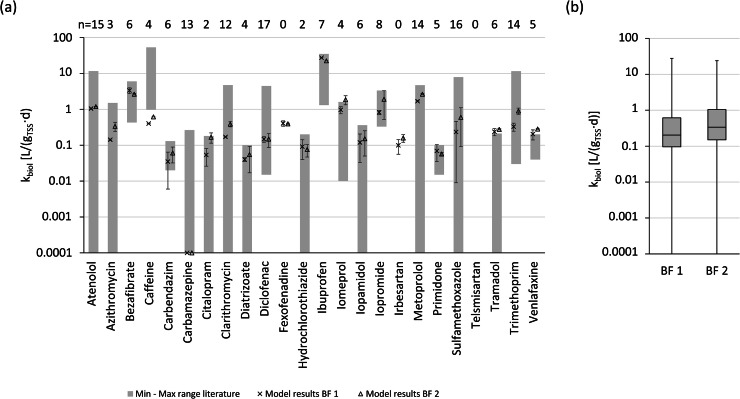
(a) k_biol_ values resulting from the parameter estimation procedure. Markers represent mean values of the twofold k_biol_ estimation (onefold for OMPs caffeine and ibuprofen) for biofilter 1 and 2 (BF 1 and BF 2). Error indicators represent the corresponding minimum and maximum value from which the mean values are calculated. Gray bars represent minimum-maximum ranges of literature values (Joss et al., 2006; Maurer et al., 2007; Majewsky et al., 2011; Wick et al., 2009; Li and Zhang, 2010; Urase and Kikuta, 2005; Abegglen et al., 2009; Plósz et al., 2010; Falås et al., 2013; Burzio et al., 2022; Wolff et al., 2021; Fernandez-Fontaina et al., 2013; Xue et al., 2010). See SI Table 25 for individual k_biol_ values from the parameter estimation procedure and additional information on literature values. The respective number of k_biol_ values from the literature is shown on top of the graph for each OMP. (b) Boxplot of the mean estimated k_biol_ values of al OMPs considered in this work for BF 1 and BF 1. Whiskers show minimum-maximum range.

When comparing the absolute values of k_biol_, no pattern was recognized that one of the biofilters showed better OMP removal kinetics (i.e. in general larger k_biol_ values) compared to the other biofilter, see Fig. 3(b). An additional 2-sample (one sample per biofilter) *t*-test for the k_biol_ values (two-sided *t*-test assuming equal variances with the null hypothesis that the two means are equal and a significance level of 0.05) yielded a p-value of 0.985. This shows that the difference in mean k_biol_ values between the two biofilters indeed was not significant. This was not necessarily to be expected given the initial inocula of the two biofilters: the inoculum of biofilter 1 must have grown in the full-scale GAC filter from which the backwash water was obtained since there was an upstream membrane filtration unit. The GAC filter from which the inoculum for biofilter 2 was obtained did not have this pretreatment, so the biomass in the backwash water could have been very similar to the suspended biomass in the conventional biological treatment step of the WWTP. Cao et al. (2022) also obtained similar average OMP biodegradation kinetics when comparing biomass from different biofilters for aWWT with different biomass compositions. The authors measured biomass as ATP, which only allows for a qualitative comparison with the results of this work. However, the lab-scale biofilters in this work were allowed to adapt for a duration of 20 batch runs before the first OMP sampling campaign started. Consequently, possible differences in initial biomass composition could have leveled out due to the similar operating conditions.

Literature ranges of k_biol_ obtained with biomass from conventional biological wastewater treatment showed a rather large degree of variation, see Fig. 3(a). There may be a number of reasons for this. For instance, the appearance of the biomass (attached or suspended) was reported to affect the value of k_biol_ (Falås et al., 2013; Wolff et al., 2021). Moreover, biomass quantification using the TSS concentration (X_TSS_) is a possible source of uncertainty, as it also includes biologically non-active particulate substances. This can systematically limit the comparability of values for k_biol_ obtained from different systems (Joss et al., 2006). X_TSS_ also fails to capture the composition of the microbial community, which is influenced by factors like sludge retention time or biofilm thickness in case of biofilm systems (Majewsky et al., 2011; Torresi et al., 2016; Wolff et al., 2021). Not appropriately considering mass transfer limitations when determining k_biol_ values is an additional source of error especially for biofilm systems (Burzio et al., 2022), but also activated sludge systems at X_TSS_ > 5 g_TSS_/L (Hatoum et al., 2019). Finally, concurrent OMP removal processes, like sorption onto biomass, volatilization (Joss et al., 2006; see Ternes et al., 2004) or abiotic chemical reactions (see Su et al., 2023), may also exacerbate this variability of k_biol_ values if not adequately accounted for. When comparing the values of k_biol_ that were determined in this work with the corresponding ranges from literature, only the k_biol_ values of the OMP caffeine were below the literature ranges, while citalopram, iomeprol, tramadol and venlafaxine had slightly higher values, see Fig. 3(a). The values of k_biol_ for the rest of the OMPs that were considered were in the range of the literature values. Interestingly, determined k_biol_ values of OMPs which were reported to be larger for biofilm processes (e.g. bezafibrate, diclofenac, iopromide and trimethoprim, see Falås et al. (2013) and Wolff et al. (2021)) were in typical ranges for suspended biomass processes (see also SI Table 25).

On the one hand, the comparison with literature values shows that the determination of k_biol_ values is not trivial. The strong simplifications of the pseudo first-order biodegradation kinetics can result in inconsistent k_biol_ values for individual OMPs, even for similar biological treatment processes. On the other hand, as this work demonstrated, the variation range can be strongly reduced by considering all relevant mass transfer processes and concurrent processes affecting OMP concentrations. No clear difference in k_biol_ values for biomass from biofilters for aWWT and from conventional biological wastewater treatment could be established. Some OMPs show larger k_biol_ values with biomass from aWWT, but this might also result from its appearance as biofilm and not necessarily from the corresponding location of the treatment processes on the WWTP. Although the k_biol_ values from biofilter 2 are difficult to interpret in this context, the values from biofilter 1 strongly support this statement due to the pretreatment step of the full-scale GAC filter from which the inoculum was obtained. In conclusion, the results of this evaluation suggest that OMP biodegradation kinetics of biomass from aWWT do not differ fundamentally from biomass from conventional biological wastewater treatment.

### 2.3 Is k_biol_ sufficient to estimate biological OMP removal in biofilters used for advanced wastewater treatment? – a conceptual evaluation based on a GAC filter

According to Eq. (1), the biological removal of individual OMPs does not only depend on k_biol_, but also on the retention time and the biomass concentration in the biofilter. Moreover, sorption of OMPs on biomass leads to a further retention in the biofilter, which decouples the hydraulic residence time from the effective contact time between OMPs and biomass (Byrns, 2001). For typical reactor setups for conventional biological wastewater treatment, Joss et al. (2006) demonstrated that OMP removal can roughly be estimated based on k_biol_. However, due to significantly different operational conditions, this is not necessarily the case for biofiltration processes used for aWWT. For this reason, this was conceptually studied using a modified version of the model used for the estimation of k_biol_ (see Section 4.4.2 for the required modifications). A GAC filter served as the basis for the geometry and operating conditions of this modified model because it represents a typical biofiltration process for aWWT. Adsorption of OMPs on the GAC was explicitly not included.

**Fig. 4:**
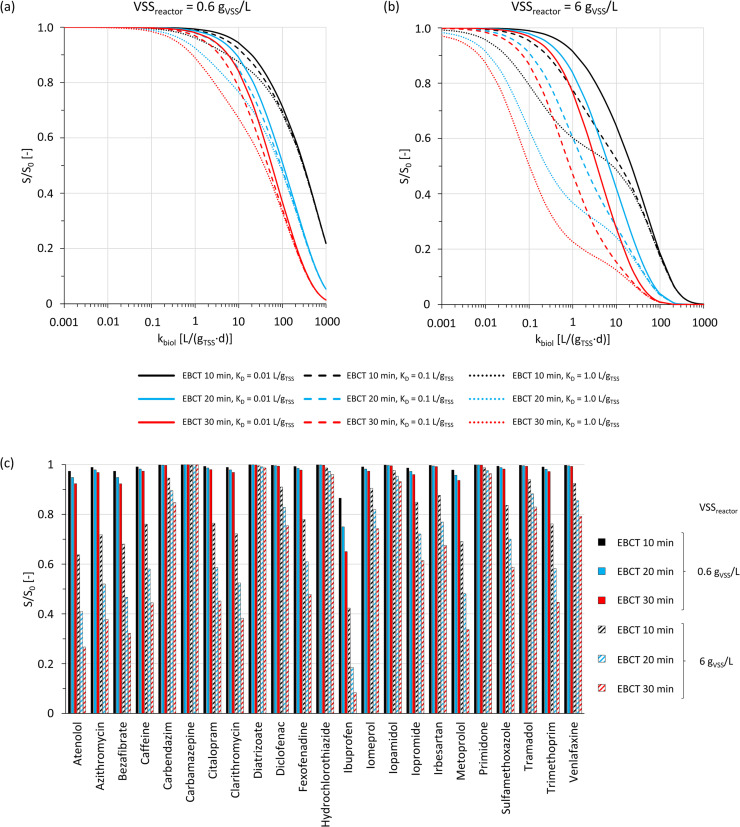
Conceptual model-based evaluation of expected biological OMP removal S/S_0_ (at steady state) in biofilters depending on the VSS concentration with regard to the reactor volume VSS_reactor_ ((a) 0.6 g_VSS_/L and (b) 6 g_VSS_/L), EBCT, water-solid distribution coefficient K_D_ and pseudo first-order biodegradation kinetic constant k_biol_. (c) expected S/S_0_ of individual OMPs during biofiltration according to mean K_D_ values from literature and k_biol_ values as determined in this work for different combinations of EBCT and VSS_reactor_. Model parameters were set to represent a GAC filter for advanced wastewater treatment (neglecting adsorptive capacity and inner porosity of the GAC).

The output of multiple runs of the modified model was processed to express predicted OMP removals (effluent concentration normalized to the corresponding influent concentration, S/S_0_) as a function of k_biol_, depending on multiple influencing factors, see Fig. 4(a) and (b). The simulation results indicate that k_biol_ alone is not sufficient to estimate biological OMP removal in biofilters. S/S_0_ was additionally strongly influenced by the model parameters empty bed contact time (EBCT), the biomass concentration (defined as mass of volatile suspended solids (VSS) per volume reactor, VSS_reactor_) and the liquid-solid partition coefficient (K_D_, i.e. sorption affinity in biomass). For instance, to reach S/S_0_ < 0.8, k_biol_ > 50 L/(g_TSS_·d) was required for conditions that do not favor biological OMP removal (short ECBT (= 10 min), small VSS_reactor_ (= 0.6 g_VSS_/L) and small K_D_ (= 0.01 L/g_TSS_)). For conditions that do favor biological OMP removal (long EBCT (= 30 min), large VSS_reactor_ (= 6 g_VSS_/L) and large K_D_ (= 1.0 g_TSS_/L)), k_biol_ > 0.02 L/(g_TSS_·d) was required to reach the same S/S_0_. Larger values for both parameters VSS_reactor_ and EBCT generally led to a decrease of predicted S/S_0_. This influence of the EBCT on biodegradation in biofilters was also reported by Terry and Summers (2018). However, this is probably only valid within the ranges of EBCT typically applied for biofilters in aWWT. A large increase in EBCT may eventually lead to a decrease of biological activity, as limitations of substrate may occur due to low filter loading rates (Paredes et al., 2016). The combined effect of K_D_ and EBCT has to be highlighted in particular. These two parameters defined the (effective) contact time between biomass and OMPs. Interestingly, the effect of the EBCT became minor and K_D_ was decisive for S/S_0_ at the lower range of k_biol_ values (lines of the same line type and therefore K_D_, but different EBCT, approach each other in Fig. 4(a) and (b)). This suggests that for OMPs showing slow biodegradation kinetics (small k_biol_), sorption to biomass and subsequent biodegradation is the relevant removal pathway. At the upper range of k_biol_, the effect of K_D_ became minor and the EBCT critical (lines of the same color and therefore EBCT, but different K_D_, approach each other in Fig. 4(a) and (b)). Thus, sorption to biomass is less relevant for OMPs showing fast biodegradation kinetics (large k_biol_) because sufficient biodegradation can take place in the dissolved state. Fig. 4(c) illustrates these rather abstract relationships by showing predicted S/S_0_ ranges for the OMPs for which k_biol_ values were estimated in the previous section of this work. A comparison of these results with OMP removals in GAC filters from literature is not straightforward due to the overlap of adsorptive and biological processes until adsorptive saturation of the GAC is reached (Simpson, 2008). Further, OMP desorption from the GAC and subsequent biodegradation may represent a symbiosis between biofilm and GAC (Herzberg et al., 2005), which has the potential to increase the overall removal of OMPs. Sbardella et al. (2018) inhibited the biomass of a lab-scale GAC filter setup and compared OMP removals to removals in a non-inhibited equivalent GAC filter. The authors showed that the contribution of biological effects to the total removal of the OMPs hydrochlorothiazide, venlafaxine and carbamazepine was < 10% whereas it was around 35% for the OMPs bezafibrate, azithromycin and sulfamethoxazole. For irbesartan, an intermediate contribution of 14% was determined. Although adsorptive saturation has not been reached during these experiments and the consequent change in concentration gradients along the GAC filter bed cannot be accounted for in the model used for our conceptual study, the results are nevertheless in qualitative agreement. In several other studies, namely Reungoat et al. (2011), Zhang et al. (2017) and Zhiteneva et al. (2021), sand filters were operated in parallel to GAC filters with the same feed and under similar operating conditions. The relative OMP removal obtained in these sand filters agreed well with the minimum-maximum ranges from Fig. 4(c) for most OMPs, see SI Figure 11. This suggests that the model yielded realistic results despite of its simplifications, thereby supporting the outcome of this conceptual evaluation. Consequently, OMP biodegradation potential expressed as k_biol_ cannot be directly linked to the relative OMP removal during biofiltration.

## 3 Conclusions

• The k_biol_ values determined in this work using the two lab-scale biofilters did not differ significantly, although it is likely that only the biomass in the inoculum for biofilter 1 grew under conditions typical for aWWT.
• Literature values of k_biol_ (of which all are referring to biomass from conventional biological wastewater treatment) agreed well with the values determined in this work. This indicates that OMP biodegradation kinetics for biomass from conventional biological wastewater treatment and aWWT are similar. This finding has a direct impact on mathematical models that focus on aWWT processes in which OMP biodegradation occurs since literature values of k_biol_ can be used in these models. However, given the large uncertainty in k_biol_ values for some OMPs due to the simplifications that are included in the pseudo first-order biodegradation kinetics, this uncertainty should be considered in the interpretation of simulation results.
• A conceptual modeling study of OMP biodegradation during biofiltration for aWWT based on the example of a GAC filter showed that k_biol_ values alone are not sufficient to even roughly estimate biological OMP removal. k_biol_ cannot be linked directly to the relative OMP removal due to the impact of additional relevant factors like the sorption affinity of OMPs to biomass, the biomass concentration in the biofilter and the EBCT on OMP removal. Consequently, the contribution of biological effects on OMP removal during aWWT is highly specific to the operational conditions of the biofiltration processes and should always be seen in this context.

## 4 Material and methods

### 4.1 Experimental setup

**Fig. 5:**
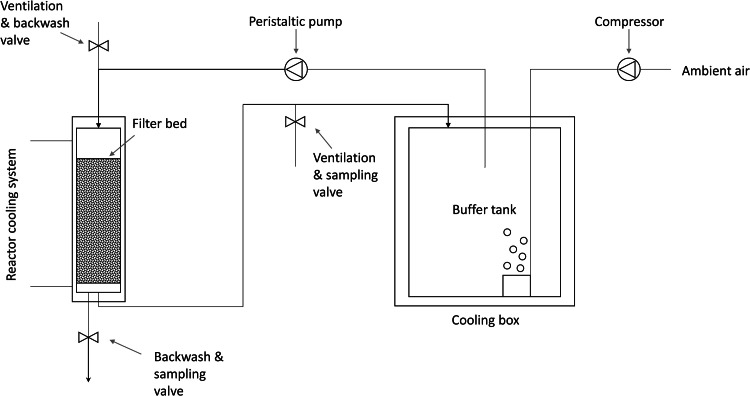
Experimental setup of one lab-scale biofilter system with recirculation of biofilter effluent.

Two lab-scale biofilter systems were operated in parallel. Each system included a biofilter and a recirculation system as displayed in Fig. 5. Each biofilter consisted of a double-walled plug-flow glass reactor (5 cm inner diameter, 40 cm height) that was filled with 0.5 L of washed and annealed expanded shale (4 - 8 mm mean grain diameter, 0.275 L displacement volume). This type of carrier material was chosen to mimic the bed porosity of a GAC filter bed, but allowed to exclude adsorption of dissolved organics which occurs in the internal pore space of the GAC (Worch, 2021). Due to the low carbon content of shale (Bucher and Grapes, 2011), it can be assumed that its adsorptive OMP removal potential is negligible (Worch, 2021). Glass frits and glass beads (0.03 L volume, 2.0 mm diameter each) were positioned at the bottom section of each reactor to keep the filter material in place. Each recirculation system included a 5 L glass buffer tank (Duran, Schott, Germany) that was kept at < 10 °C. The buffer tanks were aerated with an air flow rate of 6 L_air_/min using aeration stones and an air compressor (VP 86, VWR, France). The temperature in the biofilters was increased by using a reactor cooling system (Ecoline RE 206, Lauda, Germany) that circulated water with a temperature of 20 ± 1.5 °C in the space between the two walls of each reactor. The piping systems allowed ventilation, backwashing, sampling of the reactor effluent and emptying of the systems (excluding the reactors). Circular flow was controlled with a peristaltic pump that ensured an EBCT of 35 ± 4 min with regard to the biofilter.

The filter beds were inoculated with biomass from the backwash water of two different GAC filters (biofilter 1: full-scale GAC filter treating membrane filtrated (microfiltration 0.1 µm, Pall Corporation, Germany) effluent of WWTP 1 (see Fundneider et al., 2021b); biofilter 2: pilot-scale GAC filter treating the effluent of WWTP 2 (see Neef et al., 2022). WWTP 1 is a conventional WWTP with 75,000 population equivalents in southern Hesse (Germany) and WWTP 2 is a conventional WWTP with 725,000 population equivalents in northern Baden-Württemberg (Germany)). Backwash water was chosen as source of biomass to collect sufficient biomass for inoculation. Prior to the inoculation procedure, biomass from the two backwash waters was extracted by sieving (200 µm, Retsch, Germany), washed with the membrane-filtrated effluent from WWTP 1, thickened and stored at 4 °C. Additional information regarding the inoculums is provided in SI Table 12. Both systems were operated with the membrane-filtrated effluent from WWTP 1 (hereafter referred to as feed). For inoculation, 100 mL of each inoculum were given into the corresponding biofilter, which was then filled with feed and allowed to settle for 3 h. The purpose of this step was to promote the attachment of the biomass to the carrier material. Afterwards, the buffer tanks were filled with feed and the two systems were put into operation. The biofilters were operated in batch mode with effluent recirculation over a period of 7 days (the term batch mode was referring to the water volume in the experimental setup). This meant that the systems were emptied after 7 days (excluding the biofilters, so the pore water and the carrier material including the biofilm remained in the system), new (clean) buffer tanks were first flushed and then refilled with fresh feed. The systems were operated for a total of 193 days (or 28 batch runs). After the first batch and batch 23, the biofilters were backwashed. The backwashing procedure consisted of a stop of operation, backwashing with air and subsequent backwashing with 1.5 L of water from the previous batch's buffer tank. No third reactor system for abiotic control was operated. This was not possible due to logistical constraints and the complexity of the experimental setup. However, abiotic influencing factors were considered, as far as possible, in the model-based evaluation of the experimental results, see Section 4.4.1.

### 4.2 Sample analysis

OMP concentrations were measured for only two of the in total 28 batch runs: batch 20 (= sampling campaign 1) and batch 26 (= sampling campaign 2), resulting in two time series per sampling campaign and OMP (one for each biofilter setup). These two batch runs at the end of the operation time of the two systems were chosen to ensure that the biofilm had reached a matured state under conditions that are typical for aWWT. Prior to these two specific batches, the new (cleaned) buffer tanks were thermally pretreated at 200 °C to avoid contaminations from residues on the walls of the tanks. For batch 26, the fresh feed was additionally spiked with an OMP-mix before filling the buffer tanks (addition of 0.05 mL_OMP-mix_/L_feed_; the OMP-mix contained 10 µg/mL of each OMP marked in SI Table 1). 50 mL samples were taken from the fresh feed (before adding it to buffer tanks) and the effluent of each biofilter after 1 h, 6 h, 12 h, 24 h, 30 h, 48 h, 72 h, 96 h, 144 h and 168 h. The samples were pretreated by 0.45 µm membrane filtration and analyzed for conventional water quality parameters and OMPs. DOC was measured using a vario TOC cube (Elementar Analysesysteme, Germany). UV_254nm_ was measured with an UV–VIS spectrometer (DR 5000 Hach Lange; 50 mm QS cuvette, Hellma Analytics). PO_4_-P and NH_4_-N were measured with Hach cuvette tests (LCK 349 and LCK 304) using a Hach photometer (DR 5000). Only initial concentrations of the parameters PO_4_-P and NH_4_-N were measured for each batch. DO concentrations were measured at the effluent of biofilter 1 using an online probe (Endress und Hauser, Oxymax H). OMP concentrations were measured using High Performance Liquid Chromatography with Tandem Mass Spectrometry (HPLC-MS/MS) without sample preconcentration by the Federal Institute of Hydrology (BfG) in Koblenz, Germany. For a description of the method, the list of measured OMPs, LOQ as well as RSD, see Hermes et al. (2018).

**Table 1:** Petersen matrix describing the relevant transformation processes.

Symbol	Process	S	B	Rate expression	References
P1	Biodegradation in the liquid phase	-1		$k_{biol}\cdot X_{TSS}\cdot S$	Joss et al. (2006)
P2	Biodegradation in the sorbed phase		-1	$k_{biol}\cdot X_{TSS}\cdot B$	
P3	Sorption	-1	1	$k_{sor}\cdot X_{TSS}\cdot S$	Abegglen et al. (2009)
P4	Desorption	1	-1	$\frac{k_{sor}}{K_{D}}\cdot B$	Abegglen et al. (2009)
P5	Volatilization	-1		$H\cdot q_{G}\cdot S$	Joss et al. (2006)

### 4.3 Biomass quantification

Suspended biomass was quantified by TSS and VSS measurements which were carried out using homogenized samples and glass microfiber filters according to German standard methods (DIN, 1987). The inocula and the backwash water of the lab-scale biofilters were analyzed for TSS and VSS. After batch 28, the systems were put out of operation, emptied and the pore water was also analyzed for TSS and VSS. The mass of VSS (m_VSS_) of the biofilm was determined using German standard methods (DIN, 1985) and transferred to the mass of TSS (m_TSS_) using the VSS/TSS ratio of the pore water. Time series of m_TSS_ and m_VSS_ in the two reactors over the entire operation time of the systems were calculated using linear interpolation based on m_TSS_ and m_VSS_ initially given into the reactor with the inoculum, m_TSS_ and m_VSS_ removed during each backwash event, and m_TSS_ and m_VSS_ in each biofilter when they were put out of operation. Time series for m_TSS_ in the two biofilters for the two sampling campaigns were extracted from these two time series and used for modeling.

### 4.4 Modeling

The software AQUASIM 2.1 g (Reichert et al., 1998) was used to construct two mechanistic models. The first model described the experimental setup with biofilter effluent recirculation to the buffer tanks and was used to estimate k_biol_ values based on the experimental results. The second model described a biofilter without effluent recirculation to conceptually investigate the influence of selected operational parameters on OMP removal. Model parameters of the second model were selected to represent a GAC filter, which is a typical example of a biofiltration process for aWWT.

#### 4.4.1 Model for the estimation of k_biol_

This model considered two state variables: S represented the dissolved OMP concentrations whereas B represented the concentration of OMPs that are sorbed to the biomass. Both state variables had the unit ng/L and both were referring to the volume of the reactor compartment under consideration. Five processes influenced these state variables: Biodegradation of OMPs in the liquid phase (P1), biodegradation of OMPs in the sorbed phase (P2), sorption of OMPs to the biomass (P3), desorption of OMPs from the biomass (P4) and volatilization of OMPs (P5). The stoichiometry and reaction rates are displayed in Table 1. The model parameter k_sor_ is the sorption rate constant (L/(g_TSS_·d)) and the ratio k_sor_/K_D_ (1/d) represents the desorption rate constant with K_D_ being the solid-water distribution coefficient (L/g_TSS_). The continuous aeration of the buffer tank may have led to volatilization of OMPs. This was considered by implementing process P5 according to Joss et al. (2006). The rate expression was governed by Henry's law and assumed that phase equilibrium was reached for the rising gas bubble. Thus, the model parameter H represented the OMP-specific dimensionless Henry's law constant and q_G_ the air applied per volume of reactor and time (L_air_/(L_reactor_·d)).

**Fig. 6:**
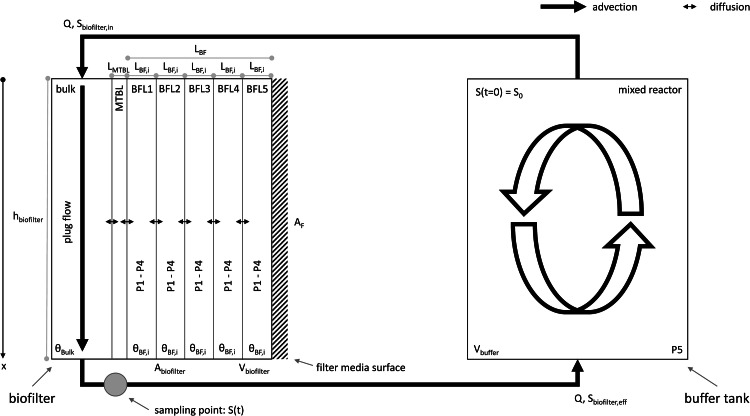
Model setup for the evaluation of the experimental results (k_biol_ estimation). The buffer tank was implemented as mixed reactor, whereas the biofilter was implemented using AQUASIM's saturated soil column compartment reactor. The biofilm was represented by 5 fully mixed layers (BFL = biofilm layer) and linked to the bulk volume by diffusion of S through a mass transfer boundary layer (MTBL). The bulk volume was modelled as plug flow with the spatial variable x describing the position in the biofilm reactor in direction of flow. Transformation processes P1–P4 took place only in the biofilm and process P5 took place only in the buffer tank.

The buffer tank was implemented as a mixed reactor whereas the biofilter was implemented using the saturated soil column compartment of AQUASIM. This reactor type was chosen because (I) it allowed to simulate advective transport in the bulk volume and (II) all processes connected to biofilm growth could be neglected. In doing so, the pseudo first-order biodegradation kinetics approach (Eq. (1)) could be easily implemented without having to consider a growth substrate and the biomass composition. Consequently, the biomass in form of m_TSS_ in the biofilter was implemented as real list variable with the operation time as corresponding argument. The TSS concentration in the biofilm (X_TSS_) was calculated by dividing m_TSS_ by the volume of the biofilm V_BF_ (see SI section 3). V_BF_ was calculated based on the external surface area of the filter bed material and an assumed biofilm thickness (see SI Section 2). For the sake of simplicity, X_TSS_ was assumed to be constant over the entire biofilter height, although it has been shown to decrease with filter bed depth (Fundneider et al., 2021b). The mobile region of the soil column compartment reactor represented the bulk volume of the filter, whereas for the biofilm five layers of equal thickness were distinguished by defining five immobile regions. Bulk and biofilm were linked by diffusive mass transfer through a mass transfer boundary layer (MTBL). The required model parameters and reactor geometry calculations are provided in SI Section 2. These model parameters were implemented as constant variables and calculated variables, respectively. The biofilm structure was assumed to be porous (without directly distinguishing between the biofilm matrix and the pore space), so both S and B were relevant in each of the biofilm layers. The processes P1 – P4 (biodegradation and (de-)sorption) were implemented to take place only in the five biofilm layers. Since aeration took place only in the buffer tank, it was assumed that volatilization was negligible in the biofilter. Consequently, process P5 was activated only in the buffer tank. The two reactors were connected by two advective links as displayed in Fig. 6. These were characterized by a flow rate Q of 0.021 m³/d, resulting in a constant EBCT of 35 min.

The following section summarizes the model assumptions by describing the mass balance equations for the different sub-systems (or sub-compartments) of the model. Advection was assumed to be the only relevant mass transfer process in direction of flow in the bulk volume of the biofilter (x-direction). Mass transfer from the bulk volume into the first biofilm layer and between the different biofilm layers was implemented as diffusion. The differential equations for the different sub-compartments within the modeled system are displayed in SI section 5. To represent Fick's first law, the exchange coefficient q_ex_ in AQUASIM had to be defined as the product of the liquid phase diffusion coefficient of the state variable under consideration with the surface area of the biofilm normalized to the reactor height, divided by the thickness of the corresponding layer. Inserting this into the mass balance equations given in the AQUASIM user manual (Reichert, 1998b) resulted in the diffusion terms as given in SI section 5. AQUASIM discretizes the x-axis of the defined biofilter reactor setup to approximate a solution for the differential equations (Reichert, 1998b). Thus, there was a spatial gradient of the state variables in the biofilm layers in x-direction, although they were defined as fully mixed reactors. However, no diffusive transfer in x-direction could take place. The input model parameters that were used are summarized in SI Table 2 to SI Table 8 and were chosen according to values from literature if no data was available.

To evaluate the influence of assumed model parameters on the biofilter effluent concentration relative to the initial concentration of the batch (S/S_0_), a local sensitivity analysis was performed using the built-in AQUASIM functionalities. The OMP specific model parameters were set to constant values (see SI Table 19). The model parameters in questions were the molecular diffusivity reduction constant for diffusion in biofilms (f_diff_), k_sor_, the biofilm thickness L_BF_, the thickness of the mass transfer boundary layer (L_MTBL_), and the initial OMP concentration in the biofilter (S_0,biofilter_). An uncertainty (expressed as the standard deviation) of 50% was chosen (see Brun et al. (2002), high degree of uncertainty) except for S_0,biofilter_ where an uncertainty of 100% was chosen. The absolute-relative sensitivity function according to Reichert (1998b) was selected as sensitivity function. AQUASIM uses model parameter values that deviate from the standard value by 1% of the selected uncertainty for the determination of the sensitivity function (Reichert, 1998a). This resulted in model parameter deviations of 0.5% or 1%, respectively, which were considered to be appropriate for the calculation of the sensitivity function. The selected model parameter uncertainties were further propagated to the uncertainty of the predicted S/S_0_ by using AQUASIM's uncertainty analysis (Reichert, 1998b). An additional local sensitivity analysis for the parameters k_biol_, K_D_ and H (see SI Table 19 for standard values; 50% standard deviation was selected) was performed in the same way to assess the importance of biodegradation, sorption and volatilization in the given experimental setup. The higher the sensitivity of S/S_0_ to one of the kinetic constants, the more important the corresponding process was assumed to be. Each sensitivity and uncertainty analysis was performed four times, since the experimental conditions between the two biofilters and the two sampling campaigns differed (i.e. biomass in the reactor). A detailed description and discussion of the results from the sensitivity analysis is provided in SI section 11. It was found that the uncertainty in the assumed biofilm thickness L_BF_ was likely the main driver of uncertainty in the predicted OMP removal. However, typical ranges of L_BF_ are available in literature (Chen et al. (2020), see Rittmann and McCarty (1980) for porous media at low substrate concentrations) and L_BF_ was assumed accordingly (see SI Table 4). The sensitivity analysis also showed that the influence of the exact value of the sorption rate constant k_sor_ was negligible for predicted OMP removal (see SI Figure 8) and thus for the estimation of k_biol_. Consequently, a uniform value for k_sor_ was chosen for all OMPs (see SI Table 6) in accordance with the literature (Abegglen et al. (2009); large value due to fast sorption process, see Ternes et al. (2004) and Torresi et al. (2017)). Moreover, the sensitivity analysis demonstrated that the two OMP-specific model parameters k_biol_ and the solid-water distribution coefficient K_D_ (determining an OMP's affinity to sorption on biomass) were not independently identifiable on the basis of the available data. Therefore, an additional estimation of K_D_ was not possible and literature values had to be used (see SI Table 18).

Subsequently, the model predictions were fitted to the experimental datasets by adjusting the model parameter k_biol_. For this purpose, the built-in parameter estimation function of AQUASIM (least-square method, see Reichert (1998b)) was used. More specifically, the secant method with a maximum number of 50 iterations was applied. OMP specific model parameters and operational conditions were modified as required depending in the corresponding dataset. The k_biol_ estimation procedure could only be performed for OMPs for which K_D_ values from literature were available (see SI Table 18) and the data quality criteria were met (i.e. 24 of the measured OMPs). This procedure yielded up to 2 results for k_biol_ per considered OMP and biofilter, depending on the quality of the individual datasets.

#### 4.4.2 Model for conceptual evaluation of influencing factors on OMP biodegradation in biofilters for aWWT

For this conceptual evaluation, a regular biofilter model was developed based on the model of the experimental setup in this work. Since GAC filters are a typical example of biofiltration processes for aWWT, model parameters describing the filter design and operational conditions were selected accordingly. Note that adsorptive removal by the GAC was explicitly neglected in this study and it was assumed that the biofilm grows on the outer surface of the GAC grains (Çeçen and Aktas, 2011; Klimenko et al., 2003; Xiaojian et al., 1991). Therefore, the inner porosity of GAC was neglected and GAC acted only as carrier material for the biofilm in this conceptual study. The following aspects of the previous model were changed in particular:

(1) The recirculation setup including the buffer tank was omitted. Thus, only the processes P1 – P4 (see Table 1) were considered in the modified model.
(2) The model parameter EBCT was implemented. The flow rate Q was expressed as a function of the biofilter reactor volume and the EBCT.
(3) The input loading of the biofilter was specified using the model parameter S_0_ as influent concentration. This also changed the boundary condition for S for the bulk volume to S(t, x = 0) = S_0_ (see SI Table 10). Note that in the non-modified model, S_0_ was the initial OMP concentration of the batch and the influent concentrations of the biofilters changed over time due to the recirculation setup.
(4) Since biomass concentrations in biofilters are frequently expressed as VSS mass per reactor volume (including the carrier material, see Fundneider et al. (2021b) and Meda (2012)), the model parameter VSS_reactor_ (VSS concentration with regard to the reactor volume) was implemented and first set to a constant value of 0.6 g_VSS_/L_reactor_ as a typical value for GAC filters (Fundneider et al., 2021b). Based on VSS_reactor_, m_TSS_ was calculated (assuming a constant VSS/TSS ratio, see SI Table 6) and used to calculate X_TSS_.
(5) The mean grain radius of the filter bed material r_g_ was set to 7.5·10^−4^ m since this is within the typical range of grain radii for GAC applications in water treatment (Fundneider et al., 2021a; van Gijn et al., 2021; Zhang et al., 2017; Zhiteneva et al., 2021).
(6) Biofilm thickness L_BF_ was set to 50 µm, which pays tribute to frequent backwashing of the GAC filters (see Chen et al. (2020)). This resulted in a VSS concentration in the biofilm of 5.6 g_VSS_/L_biofilm_ which was at the lower end of usually observed concentrations (Chen et al., 2020). f_diff_ was reduced accordingly to 0.01 (Torresi et al., 2017). The thickness of the mass transfer boundary layer L_MTBL_ was also reduced to 30 µm.

For the modified model, an additional local sensitivity analysis (absolute-relative sensitivity function, see Reichert (1998b)) was performed. The focus of this analysis lay on the sensitivity of the relative OMP removal (S/S_0_) to the model parameters S_0_, VSS_reactor_, L_BF_, L_MTBL_, r_g_ and the displacement volume of the filter bed material V_displacement_ (uncertainty of 50 % was chosen as in the non-modified model setup). It was further distinguished between a high (k_biol_ = 10 L/(g_TSS_·d)) and low (k_biol_ = 0.1 L/(g_TSS_·d)) biodegradation potential of OMPs, see Joss et al. (2006), as well as between low (K_D_ = 0.1 L/g_TSS_) and high (K_D_ = 1 L/g_TSS_) sorption affinities to biomass. For this purpose, OMP specific parameters were set according to SI Table 19 (except for k_biol_ and K_D_) and the EBCT was set constant to 20 min (Fundneider et al., 2021a). S_0,biofilter_ was set to S_0_. The rest of the model parameters remained unchanged. Predicted effluent concentrations quickly reached a steady state since biomass growth and decay were not simulated and influent concentrations were kept constant. The detailed results of the sensitivity analysis can be taken from SI section 12. It was shown that next to the biofilm thickness L_BF_, uncertainty in the biomass concentration (model parameter VSS_reactor_) is another main driver of uncertainty in predicted S/S_0_. This is critical as measurements of biomass concentrations in biofilters are rarely available. Moreover, S/S_0_ was shown to be independent of S_0_, so focusing on relative OMP removal was appropriate for this conceptual evaluation.

Based on these results, the influence of different combinations of VSS_reactor_ (0.6 g_VSS_/L_reactor_ and 6 g_VSS_/L_reactor_), EBCT (10 min, 20 min and 30 min) and K_D_ (0.01 L/g_TSS_, 0.1 L/g_TSS_ and 1 L/g_TSS_) on S/S_0_ was investigated. This involved calculating S/S_0_ for a range of k_biol_ values. Under the assumption of a constant biofilm geometry, the selected values for VSS_reactor_ correspond to VSS concentrations in the biofilm of 5.6 g_VSS_/L_biofilm_ and 55 g_VSS_/L_biofilm_. This represents the lower and upper range for typical concentrations in biofilms (Chen et al., 2020). The selected values for the EBCT were in a typical range for GAC filters used for aWWT (Fundneider et al., 2021a). The values for K_D_ were selected according to SI Figure 2 and covered the entire range of values that were reported in literature for the OMPs considered in this work. In addition, the model was used to predict S/S_0_ depending on the selected three different EBCTs and the two VSS_reactor_ for the OMPs for which values of k_biol_ were determined in the first part of this work. Thus, only OMP-specific parameters (e.g. k_biol_, K_D_) were modified. The purpose of these simulations was to show the impact of the investigated influencing factors on OMP removal in biofilters using specific examples.

## CRediT authorship contribution statement

**Tobias Kaiser:** Writing – review & editing, Writing – original draft, Visualization, Methodology, Investigation, Formal analysis, Conceptualization. **Thomas Fundneider:** Writing – review & editing, Methodology, Investigation. **Susanne Lackner:** Writing – review & editing, Supervision, Funding acquisition.

## Declaration of competing interest

The authors declare that they have no known competing financial interests or personal relationships that could have appeared to influence the work reported in this paper.

## Data Availability

Data will be made available on request

## Appendix Supplementary materials

Image, application 1
