# Supplementary material for: Biodegradation kinetics of organic micropollutants in biofilters for advanced wastewater treatment – Impact of operational conditions and biomass origin on removal
Source: Water Res X. 2024 Jul 8;24:100235. doi: 10.1016/j.wroa.2024.100235 (PMC11304067; doi:10.1016/j.wroa.2024.100235)
Supplement: Supplementary file 1 [file mmc1.pdf]

Biodegradation kinetics of organic micropollutants in biofilters for advanced wastewater treatment – impact of operational conditions and biomass origin on removal

Tobias Kaiser[1], Thomas Fundneider[1,2], Susanne Lackner[1]

[1]*Technical University of Darmstadt, Institute IWAR, Chair of Water and Environmental Biotechnology, Franziska-Braun-Straße 7, 64287 Darmstadt, Germany*

[2]*Mecana AG, Industriestrasse 39, 8864 Reichenburg, Switzerland*

Supplementary Information (SI)

Table of contents

SI section 1: Measured OMPs 3

SI section 2: Biofilter reactor geometry calculations 5

SI section 3: Kinetic model input parameters 7

SI section 4: Model input parameters for mass transfer calculations 8

SI section 5: Mass balances 9

SI section 6: Sampling campaign and biofilter specific model input parameters 11

SI section 7: Available data of conventional water quality parameters for the two sampling campaigns 12

SI section 8: OMP specific model input parameters 14

SI section 9: Measured biofilter effluent time series of relevant OMPs for the two BFs and the two SC 19

SI section 10: Model-based estimation of k_{biol} values 24

SI section 11: Outcome of the sensitivity analyses for the model describing the lab-scale biofilter setup 32

SI section 12: Outcome of the sensitivity analysis for the model used for conceptual evaluation of influencing factors on OMP biodegradation in biofilters for aWWT 34

SI section 13: Comparison of predicted ranges of S/S_0 for individual OMPs with measured S/S_0 for sand filters used for aWWT as reported in literature 36

SI section 14: List of acronyms or abbreviations used in the manuscript 37

SI nomenclature 38

References 40

SI section 1: Measured OMPs

SI Table 1: List of relevant measured organic micropollutants (OMP) including initial batch concentrations of OMPs (S_0) of sampling campaigns SC1 and SC2. LOQ stands for limit of quantification. Further information is given on whether the OMP-spike for SC2 (0.05 $mL_{OMP\text{-}mix}/L_{feed}$) contained 10 µg/mL of the respective OMP and whether measured concentrations of the respective OMP could be used for further analysis (i.e. quality criteria were met and/or sufficient data for modelling was available). For a description of the measuring method, the complete list of measured OMPs and the method's relative standard deviation, see Hermes et al. (2018).

OMP	S_0			LOQ	Spiked for SC2	Used for further analysis
	SC1	SC2				
		w/o spike	spiked			
	ng/L	ng/L	ng/L	ng/L		
Acesulfame	310	1,800	1,800	20		
Acyclovir	42	72	640	20	X	
Aliskiren	110	140	120	10		
Amisulpride	640	280	280	5		
Atenolol	87	76	680	20	X	X
Azithromycin	180	250	820	20		X
Benzotriazole	9,200	6,100	5,900	50		
Caffeine		< LOQ	600	20	X	X
Bezafibrate	50	150	810	2	X	X
Bicalutamide	7.6	5.3	5.6	1		
Candesartan	4,300	2,000	2,000	2		
Carbendazim	42	22	670	2	X	X
Carbamazepine	700	380	380	1		X
Cetirizine	150	35	35	5		
Chlorothiazide	160	95	91	2		
Citalopram	140	86	650	5	X	X
Clarithromycin	190	220	710	2	X	X
Climbazole	51	43	42	2		
Clopidogrel	3.8	5.6	5.6	1		
DEET	20	70	560	5	X	
Diatrizoate	3,500	3,500	3,500	10		X
Diclofenac	2,400	1,300	1,300	2		X
Diuron	17	15	610	1	X	
Fexofenadine	150	66	620	1	X	X
Flecainide	260	98	100	2		
Fluconazole	72	24	24	2		
Furosemide	78	53	52	50		
Gabapentin	1,800	360	320	10		
Hydrochlorothiazide	4,300	2,600	2,600	5		X
Ibuprofen	< LOQ	13	590	20	X	X
Iomeprol	11,000	15,000	15,000	50		X
Iopamidol	70	9,900	690	50	X	X
Iopromide	160	220	820	50	X	X
Irbesartan	1,200	630	650	2		X
Lidocaine	200	110	100	2		

OMP	S_0			LOQ	Spiked for SC2	Used for further analysis
	SC1	SC2				
		w/o spike	spiked			
	ng/L	ng/L	ng/L	ng/L		
Mecoprop	14	47	650	5	X	
Metoprolol	1,300	830	1,400	2	X	X
Olmesartan	440	220	220	5		
Primidone	400	190	800	5	X	X
Saccharin	60	250	940	5	X	
Sitagliptin	3,000	1,700	1,700	5		
Sulfamethoxazole	540	130	770	5	X	X
Sucralose	14,000	6,700	7,300	50		
Sulpiride	280	120	110	5		
Telmisartan	380	480	490	5		X
Terbuthylazine	< LOQ	< LOQ	620	1	X	
Terbutryn	70	11	540	1	X	
Torasemide	230	120	120	2		
Tramadol	800	400	1,000	2	X	X
Trimethoprim	220	120	720	5	X	X
Valsartan	160	1,300	1,800	2	X	
Venlafaxine	700	380	1,000	1	X	X
Xipamide	55	11	11	2		

SI section 2: Biofilter reactor geometry calculations

The saturated soil column compartment of AQUASIM (see Reichert, 1998) was used to simulate OMP removal in the lab-scale biofilters. Thus, the biofilters were represented by a combination of a (plug flow) mobile region with multiple adjacent immobile regions. The biofilm reactor compartment was not used because processes related to biofilm growth could not be represented with the available data basis. The overall objective was to keep the model as simple as possible while considering all relevant mass transfer and transformation processes of OMPs.

The following model parameters are required as input for the reactor geometry calculations. Depending on the type of model (model of the experimental system vs. conceptual biofilter model), different values may have been assumed for selected model variables to account for different operating conditions. These model parameters were implemented as constant variables in AQUASIM.

SI Table 2: Input parameters for reactor geometry calculations of the buffer tanks

Parameter	Symbol	Unit	Value	
			Experimental system	Conceptual
Buffer tank volume	V_{buffer}	L	5.0	-

SI Table 3: Input model parameters for reactor geometry calculations of the biofilters

Parameter	Symbol	Unit	Value	
			Experimental system	Conceptual
Biofilter bed volume	$V_{biofilter}$	L	0.5	0.5
Inner diameter of biofilter	$d_{biofilter}$	m	0.05	0.05
Displacement volume of the filter bed material	$V_{displacement}$	L	0.275	0.275
Mean grain radius of filter bed material	r_g	m	0.003	0.00075

SI Table 4: Assumptions for reactor geometry calculations

Parameter	Symbol	Unit	Value	
			Experimental system	Conceptual
Total biofilm thickness(4)	L_{BF}	m	0.0002(1)(5)	0.00005(3)(5)
Number of biofilm layers	n_{BF}	-	5	5
Thickness of the mass transfer boundary layer	L_{MTBL}	m	0.00005(2)(5)	0.00003(5)

(1) Takács (2022)
(2) Çeçen and Aktas (2011)
(3) under assumption of regular backwashing
(4) Assumption of constant thickness (determined by hydraulic conditions, applicable for considered short time ranges), biomass growth leads to increase of TSS concentration in biofilm volume
(5) within the valid range defined by Chen et al. (2020) and Rittmann and McCarty (1980)

The required model parameters for the description of the biofilter geometry can be calculated as displayed in SI Table 5. These model parameters were implemented as formula variables.

SI Table 5: Reactor geometry calculations as implemented in the model

Parameter	Symbol	Unit	Formula
Bed height	$h_{biofilter}$	m	$\frac{V_{biofilter}}{\pi \cdot \left(\frac{d_{biofilter}}{2}\right)^2}$
Cross sectional area of the filter bed	$A_{biofilter}$	m^2	$\frac{V_{biofilter}}{h_{biofilter}}$
Bed porosity	Θ_{bed}	-	$1 - \frac{V_{displacement}}{V_{biofilter}}$
External surface area of the filter bed material(1)	A_F	m^2	$\frac{3 \cdot V_{biofilter} \cdot (1 - \Theta_{bed})}{r_g}$
Biofilm total volume(2)	V_{BF}	m^3	$L_{BF} \cdot A_F$
Volume fraction of biofilm	Θ_{BF}	-	$\frac{V_{BF}}{V_{biofilter}}$
Volume of one biofilm layer	$V_{BF,i}$	m^3	$\frac{V_{BF}}{n_{BF}}$
Volume fraction of one biofilm layer	$\Theta_{BF,i}$	-	$\frac{V_{BF,i}}{V_{biofilter}}$
Bulk volume (biofilter)	V_{Bulk}	m^3	$V_{biofilter} \cdot \theta_{bed} - V_{BF}$
Bulk volume fraction	Θ_{Bulk}	-	$\frac{V_{Bulk}}{V_{biofilter}}$

(1) Worch (2021)
(2) Assumption: biofilm surface area is identical to filter bed material external surface area

SI section 3: Kinetic model input parameters

The rate expressions in Table 1 require specific model parameters. SI Table 6 shows their origin or how they are calculated.

SI Table 6: Kinetic model input parameters

Symbol	Name	Unit	Formula	Value	Reference
k_{biol}	Pseudo first order biodegradation constant	$L/(g_{TSS} \cdot d)$	-	Fitting target, OMP specific	this work
$X_{TSS}^{(1)}$	TSS concentration in the biofilm	g_{TSS}/L	$\frac{m_{TSS}}{V_{BF}}$	Variable, see SI Table 13 for m_{TSS}	this work
$VSS_{reactor}^{(2)}$	VSS concentration of biofilm per reactor volume (incl. carrier material)	$g_{VSS}/L_{reactor}$	-	0.6	Fundneider et al. (2021)
$VSS/TSS^{(2)}$	VSS/TSS ratio	g_{VSS}/g_{TSS}	-	0.7	this work
$X_{TSS}^{(2)}$	TSS concentration in the biofilm	g_{TSS}/L	$\frac{VSS_{reactor}}{VSS/TSS} \cdot \frac{V_{biofilter}}{V_{BF}}$	Variable	this work
k_{sor}	Sorption rate constant	$L/(g_{TSS} \cdot d)$	-	25.0	Abegglen et al. (2009)
K_D	Solid-water distribution coefficient	$L/(g_{TSS})$	-	OMP specific, see SI Table 18	-
$H^{(1)}$	Henry's law constant	-	-	OMP specific, see SI Table 17	-
$q_G^{(1)}$	Volume of air applied per volume reactor and time	$L_{air}/(L_{reactor} \cdot d)$	-	432	this work

(1) for model describing the experimental setup (with recirculation)
(2) for model used for conceptual evaluations

SI section 4: Model input parameters for mass transfer calculations

The mass transfer equations that are considered in this work require the parameters that are displayed in SI Table 7 (constant variables). On this basis, calculated variables as displayed in SI Table 8 were implemented.

SI Table 7: Model input parameters for mass transfer calculations

Symbol	Name	Unit	Formula	Value		Reference
				Experimental setup	Conceptual	
MW_{OMP} (1)	Molecular weight	g/mol	-	OMP specific, see SI Table 17	OMP specific, see SI Table 17	-
φ_{OMP} (1)	Mass density	g/cm³	-	OMP specific, see SI Table 17	OMP specific, see SI Table 17	-
f_{diff} (2)	Molecular diffusivity reduction constant for diffusion in biofilms	-	-	0.1	0.01	Torresi et al. (2017)
η	Solution viscosity	cps	-	1 (3)	1 (3)	Crittenden et al. (2012)

(1) see SI Table 17 for values
(2) depends on biofilm thickness, but was determined especially for OMP diffusion in biofilms
(3) for 20 °C and pure water

SI Table 8: Calculated model parameters for mass transfer calculations

Symbol	Name	Unit	Formula	Reference
MV_{OMP}	Molecular volume	cm³/mol	$\frac{MW_{OMP}}{\varphi_{OMP}}$	-
D_L	Liquid phase diffusion coefficient	m²/d	$\frac{13{,}26 \cdot 10^{-5}}{\eta^{1.4} \cdot MV_{OMP}^{\;0{,}589}} \cdot \frac{86{,}400\,\frac{s}{d}}{10{,}000\,\frac{m^2}{cm^2}}$	Hayduk and Laudie (1974)

SI section 5: Mass balances

The model describing the experimental system consisted of several sub-compartments. For these sub-compartments, mass balances for the two state variables S and B were defined. For the second model used for the conceptual of influencing factors on OMP biodegradation in biofilters, only SI Table 10 and SI Table 11 were relevant. Please note the additional modifications as described in the material and methods section of the paper for this model. The mass balances are based on Reichert (1998).

SI Table 9: Mass balances for the buffer tank. S and B are referring to the concentrations of both state variables in the buffer tank.

S
$$\frac{dS}{dt} = \frac{Q}{V_{buffer}} \cdot \left(S_{bulk}(t, x = h_{biofilter}) - S\right) - H \cdot q_g \cdot S$$
B
$$\frac{dB}{dt} = 0$$
Initial conditions:<br>$S(t = 0) = S_0$<br>$B(t = 0) = 0$

SI Table 10: Mass balances for the bulk volume sub-subsystem of biofilm reactor. S and B are referring to the concentrations of both state variables in the bulk volume sub-system of the biofilm reactor.

S
$$\frac{\partial S}{\partial t} = -\frac{1}{A_{biofilter} \cdot \theta_{Bulk}} \cdot \frac{\partial}{\partial x}(Q \cdot S) - D_L \cdot \frac{A_F}{L_{MTBL} \cdot V_{Bulk}} \cdot (S - S_{BF1})$$
B
$$\frac{\partial B}{\partial t} = 0$$
Initial conditions:<br>$S(t{=}0, x) = S_{0,biofilter}$<br>$B(t{=}0, x) = 0$<br><br>Boundary conditions:<br>$S(t, x{=}0) = S_{buffer\ tank} = S_{biofilter,in}$<br>$\frac{\partial^2 S(t,\ x = h_{biofilter})}{\partial x^2} = 0$<br>$Q \cdot B(t, x{=}0) = 0$<br>$\frac{\partial^2 B(t,\ x = h_{biofilter})}{\partial x^2} = 0$

SI Table 11: Mass balances for the biofilm layer k. k=1 is the biofilm layer adjacent to the bulk. S and B are referring to the concentrations of both state variables in one biofilter layer sub-system of the biofilm reactor.

S
$$\frac{dS_k}{dt} = \begin{cases} D_L \cdot \frac{A_F}{L_{MTBL} \cdot V_{BF,i}} \cdot (S_{Bulk} - S_k) - D_L \cdot f_{diff} \cdot \frac{A_F}{L_{BF}/n_{BF} \cdot V_{BF,i}} \cdot (S_k - S_{k+1}) - k_{biol} \cdot X_{TSS} \cdot S_k - k_{sor} \cdot X_{TSS} \cdot S_k + \frac{k_{sor}}{K_D} \cdot B_k & for\ k = 1 \\ D_L \cdot f_{diff} \cdot \frac{A_F}{L_{BF}/n_{BF} \cdot V_{BF,i}} \cdot (S_{k-1} - S_k) - D_L \cdot f_{diff} \cdot \frac{A_F}{L_{BF}/n_{BF} \cdot V_{BF,i}} \cdot (S_k - S_{k+1}) - k_{biol} \cdot X_{TSS} \cdot S_k - k_{sor} \cdot X_{TSS} \cdot S_k + \frac{k_{sor}}{K_D} \cdot B_k & for\ 1 < k < n_{BF} \\ D_L \cdot f_{diff} \cdot \frac{A_F}{L_{BF}/n_{BF} \cdot V_{BF,i}} \cdot (S_{k-1} - S_k) - k_{biol} \cdot X_{TSS} \cdot S_k - k_{sor} \cdot X_{TSS} \cdot S_k + \frac{k_{sor}}{K_D} \cdot B_k & for\ k = n_{BF} \end{cases}$$
B
$$\frac{dB_k}{dt} = -k_{biol} \cdot X_{TSS} \cdot B_k + k_{sor} \cdot X_{TSS} \cdot S_k - \frac{k_{sor}}{K_D} \cdot B_k \quad for\ 1 \leq k \leq n_{BF}$$
Initial conditions:<br>$S_k(t=0) = S_{0,biofilter}$ for $1 \leq k \leq n_{BF}$<br>$B_k(t=0) = K_D \cdot X_{TSS} \cdot S_k$ for $1 \leq k \leq n_{BF}$

SI section 6: Sampling campaign and biofilter specific model input parameters

The absolute mass of TSS (m_{TSS}) in the reactor was specific to both the sampling campaign and the biofilter. SI Table 12 shows the characteristics of the inoculum of the two biofilters. SI Table 13 shows the time series of m_{TSS} for each sampling campaign that were determined based on available TSS measurements and linear interpolation.

SI Table 12: Characteristics of GAC filter backwash water prepared for inoculation of the two biofilters

Name	Symbol	Unit	Biofilter 1$^{(1)}$	Biofilter 2$^{(2)}$
Total suspended solids	X_{TSS}	g_{TSS}/L	12.62	7.47
Volatile suspended solids	X_{VSS}	g_{VSS}/L	7.47	5.36
VSS/TSS ratio	VSS/TSS	g_{VSS}/g_{TSS}	0.59	0.72

(1) prepared backwash water from GAC filter on WWTP 1 (Fundneider et al., 2021) (a conventional WWTP in southern Hesse (Germany) with 75,000 population equivalents)
(2) prepared backwash water from GAC filter on WWTP 2 (Neef et al., 2022) (a conventional WWTP with 725,000 population equivalents in northern Baden-Württemberg (Germany))

SI Table 13: Interpolated time series of the biomasses for the two biofilters (BF) and two sampling campaigns (SC). The Time column refers to the start of the respective batch run.

Time	BF 1		BF 2	
	SC1	SC2	SC1	SC2
	m_{TSS}			
d	g			
0	1.423	0.691	0.815	0.552
1	1.424	0.693	0.816	0.553
2	1.426	0.694	0.816	0.553
3	1.427	0.695	0.817	0.554
4	1.428	0.697	0.818	0.554
5	1.430	0.698	0.818	0.555
6	1.431	0.699	0.819	0.556
7	1.432	0.701	0.819	0.556

SI section 7: Available data of conventional water quality parameters for the two sampling campaigns

For the nutrients NH_4-N and PO_4-P, only initial concentrations for the two sampling campaigns were measured. Results are displayed in SI Table 14. For the parameters UV_{254nm} and DOC, time series over the duration of the sampling campaigns were measured (see SI Table 15). Dissolved oxygen measurements in the effluent of biofilter 1 can be taken from SI Table 16.

SI Table 14: Initial concentrations of nutrients NH_4-N and PO_4-P for the two sampling campaigns (SC). Limit of quantification (LOQ) for NH_4-N measurements was 0.015 mg NH_4-N/L.

Parameter	Unit	SC1	SC2
NH_4-N	mg/L	< LOQ	< LOQ
PO_4-P	mg/L	0.17	0.098

SI Table 15: Measured UV_{254nm} and DOC concentration datasets. Time refers to the start of the respective batch (i.e. sampling campaign SC).

Time	Biofilter 1				Biofilter 2			
	SC1		SC2		SC1		SC2	
	UV_{254nm}	DOC	UV_{254nm}	DOC	UV_{254nm}	DOC	UV_{254nm}	DOC
d	1/m	mg/L	1/m	mg/L	1/m	mg/L	1/m	mg/L
0	13.37	6.21	8.4	4.10	13.37	6.21	8.4	4.10
0.042	12.65	6.22	8.49	4.20	13.03	6.37	8.39	4.31
0.25	12.51	6.17	8.26	4.17	12.93	6.33	8.25	4.18
0.5	12.32	6.09	8.16	4.04	12.79	6.27	8.15	4.09
1	11.95	5.95			12.56	6.18		
1.25			8.06	4.02			8.1	4.08
2	11.63	5.83	7.92	3.77	12.32	6.09	8	3.81
3	11.29	5.69	7.64	3.81	12.32	6.09	7.75	4.03
4	11.08	5.61	7.6	3.64	11.93	5.94	7.66	3.70
6	10.75	5.48	7.41	3.48	11.64	5.83	7.52	3.60
7	10.61	5.43	7.33	3.49	11.54	5.79	7.58	3.61

SI Table 16: Dissolved oxygen (DO) datasets for the two sampling campaigns (SC) for the effluent of biofilter 1. Time refers to the start of the respective batch run.

SC1		SC2	
Time	DO	Time	DO
d	mg/L	d	mg/L
0.00	6.27	0.00	7.18
0.03	6.78	0.21	7.41
0.11	6.93	0.46	6.33
0.24	7.41	0.96	6.39
0.49	7.16	1.96	
0.99	6.42	2.96	
1.11	6.83	3.96	
1.24	7.03	5.96	5.91
1.99	6.47	6.96	6.15
2.24	7.64		
2.99	6.36		
3.99	6.26		
6.41	6		
6.99	5.72		

SI section 8: OMP specific model input parameters

SI Table 17: OMP-specific model parameters for measured OMPs (except K_D and k_{biol}) that were used in both models

OMP	MW[1]	φ_{OMP}[2]	H
	g/mol	g/cm³	-
Atenolol	266.34	1.1	5.6E-17[3]
Azithromycin	749.0	1.2	4.0E-25[4]
Bezafibrate	361.82	1.3	1.06E-09[3]
Caffeine	194.02	1.5	2.9E-11[3]
Carbendazim	191.2	1.5	6.1E-11[4]
Carbamazepine	236.3	1.3	6.3E-08[3]
Citalopram	324.4	1.2	1.1E-09[3]
Clarithromycin	788.0	1.2	4.0E-25[5]
Diatrizoate	613.9	2.6	7.4E-17[4]
Diclofenac	296.1	1.4	2.2E-7[3]
Fexofenadine	501.7	1.2	6.9E-11[6]
Hydrochlorothiazide	297.8	1.7	2.2E-12[3]
Ibuprofen	206.3	1.0	5.0E-5[3]
Iomeprol	777.1	2.3	5.0E-24[7]
Iopamidol	777.1	2.3	5.0E-24[4]
Iopromide	791.1	2.2	4.0E-25[8]
Irbesartan	428.5	1.3	4.7E-13[3]
Metoprolol	267.36	1.0	8.7E-10[3]
Primidone	218.3	1.2	6.6E-13[3]
Sulfamethoxazole	253.3	1.5	1.1E-11[3]
Telmisartan	514.6	1.2	9.5E-15[3]
Tramadol	263.4	1.0	5.6E-09[3]
Trimethoprim	290.3	1.3	5.0E-11[3]
Venlafaxine	277.4	1.1	1.4E-08[3]

(1) http://www.chemspider.com/
(2) http://www.chemspider.com/ (predicted data using the ACD/Labs Percepta Platform – PhysChem Module)
(3) http://www.chemspider.com/ (predicted data using the US EPA's EPISuite™) and transformed into the dimensionless form using the EPA On-line Tools for Site Assessment Calculations (https://www3.epa.gov/ceampubl/learn2model/part-two/onsite/henryslaw.html)
(4) https://pubchem.ncbi.nlm.nih.gov/ and transformed into the dimensionless form using the EPA On-line Tools for Site Assessment Calculations (https://www3.epa.gov/ceampubl/learn2model/part-two/onsite/henryslaw.html)
(5) https://webetox.uba.de/webETOX/public/basics/literatur/download.do;jsessionid=C127F6C4F768D5FC28FC82DA6D7C290B?id=11 and transformed into the dimensionless form using the EPA On-line Tools for Site Assessment Calculations (https://www3.epa.gov/ceampubl/learn2model/part-two/onsite/henryslaw.html)
(6) assumed to be similar to terfenadine, obtained according to (3)
(7) assumed to be similar to iopamidol, obtained according to (3)
(8) https://www.parchem.com/chemical-supplier-distributor/Iopromide-131580.aspx and transformed into the dimensionless form using the EPA On-line Tools for Site Assessment Calculations (https://www3.epa.gov/ceampubl/learn2model/part-two/onsite/henryslaw.html)

SI Table 18: K_D values from literature review. The mean value was used for modeling. Min-max ranges include uncertainty reported with the literature values.

OMP	K_D Mean[21] (L/g$_{TSS}$)	K_D Min (L/g$_{TSS}$)	K_D Max (L/g$_{TSS}$)	Literature values
Atenolol	0.464	0	3.453	< 0.03[1], 0.035[2], 0.064 ± 0.088[3], 0.0059 ± 0.0044[4], 0.040 ± 0.050[5], 0.038 ± 0.033[6], 1.6 ± 0.2[9], 2.36 ± 1.093[10], 0.0[11]
Azithromycin	0.888	0.290	1.8	1.4 ± 0.4[8], 0.376 ± 0.086[13]
Bezafibrate	0.069	0	0.098	< 0.05[7], 0.087 ± 0.011[8], 0.087 ± 0.011[7], < 0.05[8],
Caffeine	0.257	0	0.71	< 0.03[1],[2], 0.71[19], 0.80[20]
Carbendazim	0.27	0	0.72	0.27 ± 0.45[12]
Carbamazepine	0.190	0	0.940	0.135 ± 0.039[3], 0.194 ± 0.094[4], 0.164 ± 0.049[5], 0.017 ± 0.001[6], < 0.008[7], < 0.075[8], 0.0012 ± 0.0005[14], 0.034[15], 0.089 ± 0.002[16], < 0.003[17], < 0.001[18], 0.94[19], 0.81[20]
Citalopram	1.22	1.117	1.323	1.22 ± 0.103[10]
Clarithromycin	0.731	0.169	1.360	0.73 ± 0.05[7], 1.2 ± 0.16[8], 0.262 ± 0.093[13]
Diatrizoate	0.007	0	0.007	< 0.007[7]
Diclofenac	0.161	0.013	1.2	< 0.03[1],[2], 0.118 ± 0.095[3], 0.194 ± 0.255[4], 0.321 ± 0.402[5], 0.8 ± 0.4[9], 0.016 ± 0.003[14], 0.016[15], 0.151 ± 0.002[16], 0.079 ± 0.031[17], 0.032 ± 0.014[18], 0.10[19], 0.20[20]
Fexofenadine	0.27	0.13	0.41	0.27 ± 0.14[9]
Hydrochlorothiazide	0.022	0.017	0.036	0.0202 ± 0.0034[3], 0.0235 ± 0.0126[4], 0.0223 ± 0.0125[5]
Ibuprofen	0.049	0	0.211	< 0.03[1],[2], 0.0[3],[4], 0.05 ± 0.018[7], 0.006 ± 0.004[8], 0.0071 ± 0.002[14], 0.072[15], 0.209 ± 0.002[16], 0.112 ± 0.070[17], 0.024 ± 0.005[18]
Iomeprol	0.006	0.001	0.010	< 0.01[7], 0.001 ± 0.00001[8]
Iopamidol	0.01	0	0.01	< 0.01[7]
Iopromide	0.011	0	0.044	< 0.007[7], 0.014 ± 0.03[8], 0.011 ± 0.001[14]
Irbesartan	0.3	0	0.8	0.3 ± 0.5[9]
Metoprolol	0.099	0.01	0.25	0.065[6], 0.18 ± 0.03[10], 0.23 ± 0.02[11], 0.01[19],[20]
Primidone	0.029	0.006	0.06	< 0.03[1],[2], 0.007 ± 0.001[6], 0.05 ± 0.01[8]

OMP	K_D Mean[21]	Min	Max	Literature values
	L/g_{TSS}			
Sulfamethoxazole	0.100	0.003	0.400	< 0.03[1],[2], 0.077 ± 0.060[3], 0.060 ± 0.049[4], 0.063 ± 0.042[5], 0.04 ± 0.013[7], 0.05 ± 0.013[8], 0.3 ± 0.1[9], 0.256 ± 0.169[13], 0.269 ± 0.002[16], 0.009 ± 0.006[17], 0.011 ± 0.007[18]
Telmisartan	0.8	0.4	1.2	0.8 ± 0.4[9]
Tramadol	0.047	0.046	0.048	0.047 ± 0.001[6]
Trimethoprim	0.204	0.016	0.42	0.119 ± 0.049[1], 0.193 ± 0.104[2], 0.253 ± 0.037[3], 0.225 ± 0.087[4], 0.320 ± 0.117[5], 0.33 ± 0.025[8], 0.35 ± 0.07[9], 0.208 ± 0.049[13], 0.200 ± 0.001[16], 0.025 ± 0.008[17], 0.025 ± 0.009[18], 0.3[19]
Venlafaxine	0.070	0	0.13	0.05 ± 0.06[9], 0.09 ± 0.04[10]

Sludge types:
(1) secondary sludge from a CAS plant (SRT 5 d, inactivation: lyophilization and dry-heat inactivation technique), Stevens-Garmon et al. (2011)
(2) secondary sludge from a CAS plant (SRT 6 d, inactivation: lyophilization and dry-heat inactivation technique), Stevens-Garmon et al. (2011)
(3) secondary sludge from a CAS plant, Radjenović et al. (2009)
(4) secondary sludge from a MBR plant, Radjenović et al. (2009)
(5) secondary sludge from a MBR plant, Radjenović et al. (2009)
(6) secondary sludge from a CAS plant (SRT 18 d, inactivation: chemical (sodium azide)), Wick et al. (2009)
(7) secondary sludge from a MBR plant (single house plant, without primary clarification) (SRT > 150 d), Abegglen et al. (2009)
(8) secondary sludge from a MBR plant (single house plant) (SRT > 100 d), Abegglen et al. (2009)
(9) secondary sludge from CAS plant (SRT 10 d, inactivation: chemical with sodium sulfite), Hörsing et al. (2011)
(10) biofilm on carriers from a laboratory-scale nitrifying MBBR fed with spiked (ammonium and phosphate) effluent wastewater from a CAS plant (inactivation: chemically with allylthiourea and sodium azide), mean of three values with biofilm thicknesses of 50 μm, 200 μm and 500 μm, Torresi et al. (2017)
(11) secondary sludge from a CAS plant, enriched in nitrifying bacteria using a SBR (inactivation: dried at 90 ± 5 °C for 15 days), Sathyamoorthy et al. (2013)
(12) secondary sludge from a CAS plant (stored at -20°C until analysis, calculated using mass balance of entire plant), Kupper et al. (2006)
(13) secondary sludge from a CAS plant (calculated as ratio of sorbed and dissolved fraction measured), Göbel et al. (2005)
(14) secondary sludge from a CAS plant (directly aerated with argon to minimize oxygenic degradation processes), Ternes et al. (2004)
(15) secondary sludge from a CAS plant (stored in a reactor with synthetic wastewater, low DOC during experiment), Urase and Kikuta (2005)
(16) secondary sludge from CAS plants, values represent mean of 9 sludges, Hyland et al. (2012)
(17) secondary sludge from a pilot-scale MBR, obtained from simulation of the MBR, Fernandez-Fontaina et al. (2013)
(18) secondary sludge from a pilot scale MBR, obtained from simulation in a batch reactor setup, Fernandez-Fontaina et al. (2013)
(19) secondary sludge from an A^2/O-MBR plant (oxic tank), Xue et al. (2010)
(20) secondary sludge from an A^2/O-MBR plant (membrane tank), Xue et al. (2010)
Further information:
(21) if only upper threshold value is given, this value was used

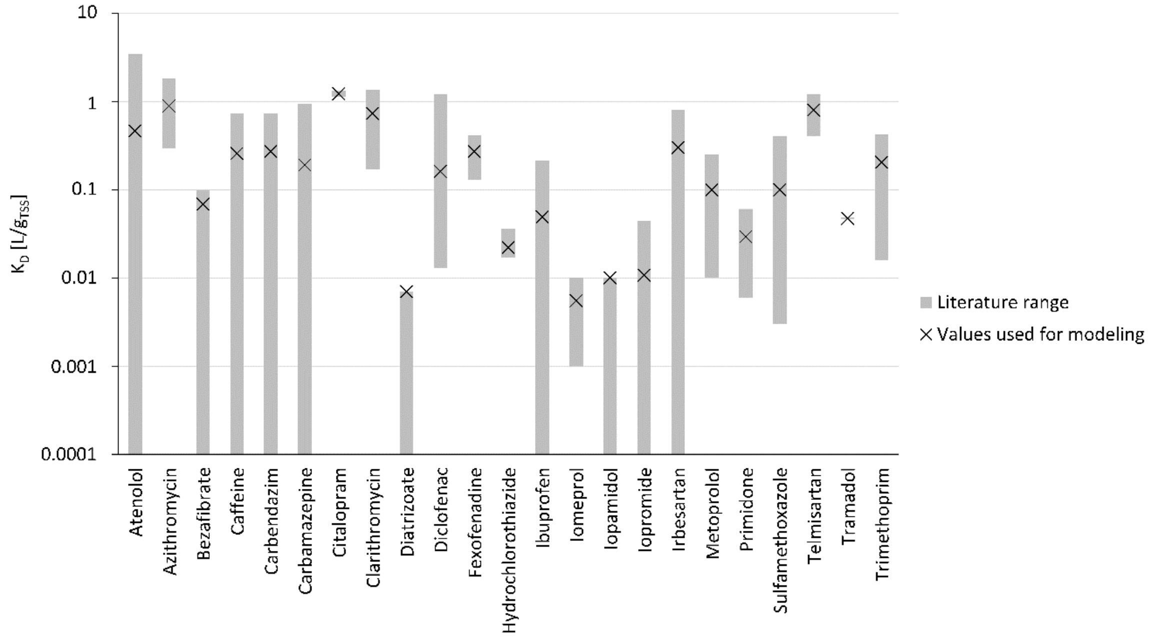

SI Figure 1: K_D value ranges from literature review and mean K_D values that were used for modeling

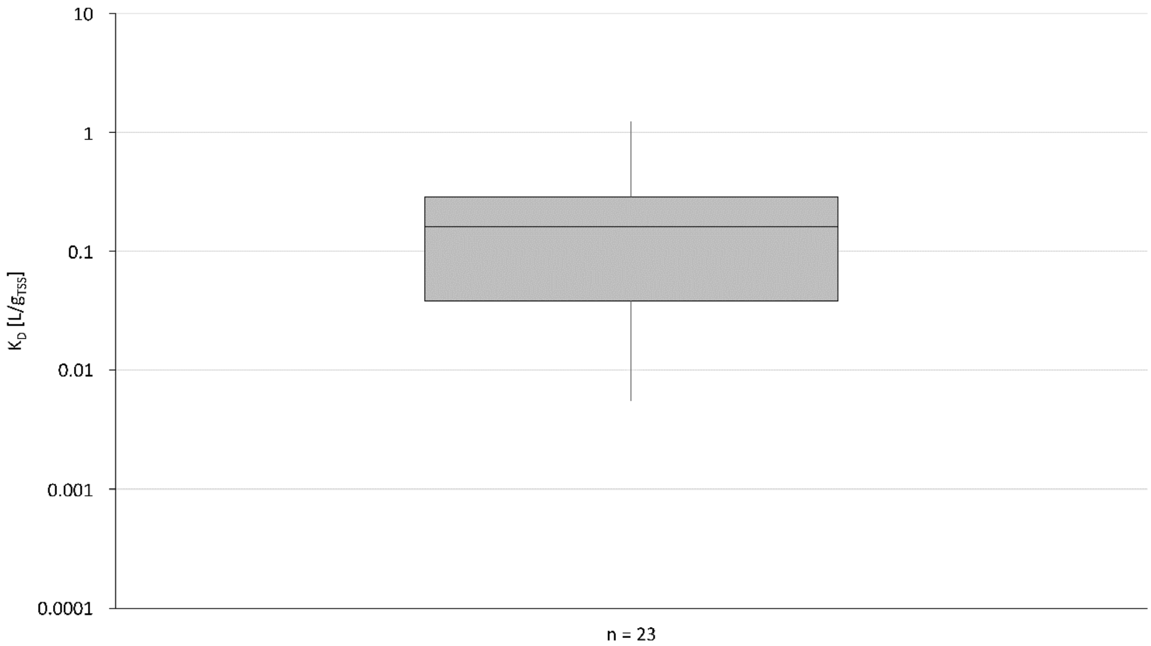

SI Figure 2: Boxplot of mean K_D values of all considered OMPs. Whiskers are showing the minimum-maximum range over all considered OMPs.

SI Table 19: Selected values for OMP-specific model input parameters used for the sensitivity analysis

Name	Symbol	Unit	Value
Dimensionless Henry's law constant	H	-	$2.0 \cdot 10^{-11}$
Pseudo first-order biodegradation constant	k_{biol}	$L/(g_{TSS} \cdot d)$	0.5
Solid-water distribution coefficient	K_D	L/g_{TSS}	0.1
OMP molar volume	MV	cm^3/mol	300
Initial OMP concentration in the buffer tank	S_0	ng/L	1,000
Initial OMP concentration in the filter bed	$S_{0,biofilter}$	ng/L	1,000

SI section 9: Measured biofilter effluent time series of relevant OMPs for the two BFs and the two SC

SI Table 20: Time series of measured biofilter effluent concentrations of relevant OMPs for the two sampling campaigns (SC) and the two biofilters (BF)

		Time	Acesulfame	Acyclovir	Aliskiren	Amisulpride	Atenolol	Azithromycin	Benzotriazole	Caffeine	Bezafibrate	Bicalutamide	Candesartan	Carbendazim
		d	ng/L	ng/L	ng/L	ng/L	ng/L	ng/L	ng/L	ng/L	ng/L	ng/L	ng/L	ng/L
SC 1	BF 1	0.042	290	31	71	590	52	130	7,500	< LOQ	29	12	3,800	40
		0.25	290	39	66	570	37	130	7,200	< LOQ	25	15	3,900	39
		0.5	290	26	56	540	26	100	6,600	< LOQ	16	12	3,900	36
		1	290	< LOQ	48	540	< LOQ	78	5,900	< LOQ	6	16	3,900	39
		2	290	27	43	460	< LOQ	50	4,500	< LOQ	< LOQ	12	3,800	35
		3	270	< LOQ	29	440	< LOQ	22	3,300	< LOQ	< LOQ	8.8	3,700	37
		4	290	< LOQ	27	440	< LOQ	< LOQ	2,900	< LOQ	< LOQ	9.2	3,700	38
		6	290	< LOQ	21	340	< LOQ	< LOQ	1,800	< LOQ	< LOQ	20	3,800	36
		7	280	< LOQ	18	320	< LOQ	< LOQ	1,500	< LOQ	< LOQ	9.9	3,500	31
	BF 2	0.042	300	25	79	670	67	140	8,800	< LOQ	42	15	3,800	39
		0.25	290	24	78	630	55	160	9,000	< LOQ	37	15	3,700	36
		0.5	300	30	77	620	42	130	8,600	< LOQ	32	12	3,800	36
		1	300	< LOQ	57	630	28	100	9,000	< LOQ	24	12	3,900	37
		2	300	< LOQ	50	600	< LOQ	73	8,500	< LOQ	9.3	15	3,800	34
		3	290	< LOQ	45	580	< LOQ	42	8,300	< LOQ	3.6	10	3,800	36
		4	290	< LOQ	42	560	< LOQ	27	7,100	< LOQ	< LOQ	12	3,800	37
		6	300	< LOQ	27	530	< LOQ	< LOQ	7,400	< LOQ	< LOQ	14	3,700	33
		7	300	< LOQ	33	560	< LOQ	< LOQ	7,400	< LOQ	< LOQ	14	3,800	32
SC 2	BF 1	0.042	1,600	490	150	270	480	700	5,500	490	630	11	2,000	550
		0.25	1,700	490	130	260	470	770	5,500	530	660	11	2,000	600
		0.5	1,700	490	140	260	460	660	5,500	520	610	16	2,000	560
		1.25	1,600	460	120	260	300	540	5,100	470	450	12	2,000	570
		2	1,700	410	130	260	120	470	4,900	370	160	10	1,900	560
		3	1,700	290	120	260	48	420	4,700	280	32	13	2,000	550
		4	1,700	260	110	250	22	290	4,200	230	5.3	14	2,000	540
		6	1,600	160	110	230	< LOQ	210	3,500	130	< LOQ	13	2,000	490
		7	1,600	140	91	220	< LOQ	160	3,500	96	< LOQ	15	2,000	490
	BF 2	0.042	1,700	490	160	270	590	760	5,800	550	710	12	2,000	610
		0.25	1,700	480	140	270	520	750	5,700	530	700	9.3	2,100	580
		0.5	1,800	560	130	290	490	510	5,800	550	680	12	2,100	640
		1.25	1,700	450	120	290	350	550	5,700	460	570	9.9	2,100	600
		2	1,700	330	120	270	150	320	5,600	2200(1)	260	12	2,100	580
		3	1,700	180	110	270	64	240	5,200	260	66	13	2,000	550
		4	1,700	160	100	270	37	170	4,800	230	18	13	2,000	540
		6	1,700	97	96	270	< LOQ	91	4,500	130	< LOQ	16	2,100	500
		7	1,700	64	75	260	< LOQ	66	4,400	100	< LOQ	15	2,000	490

(1) outlier, was omitted

SI Table 21: Time series of measured biofilter effluent concentrations of relevant OMPs for the two sampling campaigns (SC) and the two biofilters (BF)

		Time	Carbamazepine	Cetirizine	Chlorothiazide	Citalopram	Clarithromycin	Climbazole	Clopidogrel	DEET	Diatrizoate	Diclofenac	Diuron	Fexofenadine
		d	ng/L	ng/L	ng/L	ng/L	ng/L	ng/L	ng/L	ng/L	ng/L	ng/L	ng/L	ng/L
SC 1	BF 2	0.042	640	140	140	110	140	40	2.4	24	3,200	2,200	17	100
		0.25	670	140	150	87	130	44	2.6	25	3,200	2,100	18	93
		0.5	660	140	140	93	120	36	1.9	27	3,200	2,000	17	79
		1	680	140	160	85	80	32	1.3	24	3,100	1,900	18	59
		2	680	130	170	67	48	21	< LOQ	26	3,200	1,600	17	35
		3	650	120	170	58	23	11	< LOQ	26	2,900	1,300	16	21
		4	700	110	190	52	15	8.1	< LOQ	30	3,100	1,200	16	14
		6	690	95	220	36	4.1	4.8	< LOQ	30	3,200	760	15	5.3
		7	670	93	240	35	2.7	3.5	< LOQ	28	3,100	650	14	3.7
	BF 2	0.042	700	150	140	110	150	47	2.9	19	3,400	2,300	16	130
		0.25	700	140	140	85	170	49	2.5	20	3,400	2,300	16	130
		0.5	710	140	140	100	130	46	1.9	19	3,400	2,300	17	120
		1	720	150	150	84	100	39	1.3	20	3,400	2,300	17	110
		2	690	140	150	69	66	32	0.6	20	3,300	2,100	17	82
		3	690	130	160	55	39	25	< LOQ	19	3,300	2,000	16	63
		4	690	120	170	40	26	19	< LOQ	20	3,200	1,900	15	47
		6	700	290	190	20	6.1	11	< LOQ	23	3,300	1,600	14	24
		7	700	110	190	19	5.5	9.8	< LOQ	23	3,300	1,500	13	22
SC 2	BF 1	0.042	370	38	97	500	650	33	4.1	480	3,200	1,200	300	540
		0.25	390	37	98	500	610	35	3.9	500	3,300	1,300	420	540
		0.5	390	37	94	530	610	34	4.2	480	3,300	1,200	420	520
		1.25	380	36	93	390	540	29	2.5	470	3,200	1,200	440	470
		2	380	36	100	370	420	20	0.9	460	3,200	1,100	400	390
		3	390	37	100	300	340	15	0.8	450	3,300	990	410	330
		4	370	36	110	280	270	12	0.6	430	3,300	920	380	260
		6	380	33	110	300	180	7.2	< LOQ	390	3,200	760	350	180
		7	380	32	120	200	130	5.6	< LOQ	360	3,300	690	370	160
	BF 2	0.042	390	38	100	570	650	35	4.4	510	3,200	1,300	430	540
		0.25	390	37	94	510	580	36	3.5	500	3,200	1,300	490	540
		0.5	390	39	99	530	530	33	3.6	510	3,300	1,300	510	540
		1.25	390	36	100	340	500	33	1.7	500	3,200	1,300	510	510
		2	390	36	100	330	330	23	0.9	480	3,200	1,200	460	410
		3	390	37	110	230	220	18	0.5	460	3,200	1,000	430	350
		4	380	34	110	210	170	15	< LOQ	450	3,200	960	400	310
		6	390	34	110	170	82	9.6	< LOQ	430	3,200	810	380	230
		7	400	31	120	110	57	8.1	< LOQ	400	3,100	720	360	190

SI Table 22: Time series of measured biofilter effluent concentrations of relevant OMPs for the two sampling campaigns (SC) and the two biofilters (BF)

		Time	Flecainide	Fluconazole	Furosemide	Gabapentin	Hydrochlorothiazide	Ibuprofen	Iomeprol	Iopamidol	Iopromide	Irbesartan	Lidocaine	Mecoprop
		d	ng/L	ng/L	ng/L	ng/L	ng/L	ng/L	ng/L	ng/L	ng/L	ng/L	ng/L	ng/L
SC 1	BF 1	0.042	250	68	76	1,400	4,100	< LOQ	8,500	53	180	1,100	170	13
		0.25	230	68	78	1,400	4,600	< LOQ	8,300	67	110	1,000	160	14
		0.5	220	67	76	1,400	4,400	< LOQ	7,900	64	130	1,000	160	12
		1	220	69	65	1,100	4,000	< LOQ	6,900	74	120	940	150	12
		2	200	70	55	710	3,900	< LOQ	5,100	73	82	830	140	11
		3	200	60	< LOQ	470	3,600	< LOQ	3,700	63	59	700	130	8.8
		4	190	69	< LOQ	320	4,000	< LOQ	2,900	53	77	670	130	8.8
		6	180	66	< LOQ	170	3,600	< LOQ	1,400	< LOQ	< LOQ	500	110	5.3
		7	180	66	< LOQ	170	3,600	< LOQ	1,300	65	< LOQ	420	100	< LOQ
	BF 2	0.042	240	70	69	1,600	4,500	< LOQ	11,000	80	150	1,100	180	15
		0.25	250	68	71	1,500	4,500	< LOQ	9,800	56	130	1,000	170	13
		0.5	250	67	70	1,400	4,400	< LOQ	9,500	94	150	1,100	160	14
		1	250	70	66	1,300	4,100	< LOQ	8,200	58	170	1,100	160	13
		2	230	70	59	860	4,000	< LOQ	6,700	58	140	930	160	13
		3	210	67	< LOQ	540	4,100	< LOQ	5,100	67	96	830	150	11
		4	190	67	< LOQ	350	3,800	< LOQ	4,200	69	95	750	140	10
		6	180	69	< LOQ	190	3,900	< LOQ	2,300	55	< LOQ	540	130	8.1
		7	180	71	< LOQ	170	3,700	< LOQ	2,300	67	< LOQ	520	130	8
SC 2	BF 1	0.042	150	25	< LOQ	300	2,500	290	13,000	530	650	630	120	540
		0.25	150	25	< LOQ	280	2,500	170	14,000	600	650	590	110	570
		0.5	150	25	< LOQ	260	2,500	83	13,000	560	710	590	110	570
		1.25	140	23	< LOQ	250	2,500	11	12,000	610	710	540	100	540
		2	130	24	< LOQ	200	2,400	< LOQ	11,000	620	600	510	96	520
		3	130	25	< LOQ	180	2,300	< LOQ	11,000	550	520	460	95	500
		4	120	23	< LOQ	160	2,300	< LOQ	9,300	600	510	420	95	450
		6	130	25	< LOQ	130	2,100	< LOQ	7,300	540	280	340	91	380
		7	110	25	< LOQ	110	2,000	< LOQ	6,500	560	250	310	88	350
	BF 2	0.042	140	26	< LOQ	310	2,700	320	13,000	570	770	610	120	590
		0.25	150	26	< LOQ	320	2,500	230	13,000	600	650	620	110	580
		0.5	130	26	< LOQ	330	2,700	150	13,000	630	610	580	110	600
		1.25	130	25	< LOQ	330	2,600	47	12,000	570	500	560	100	590
		2	140	25	< LOQ	260	2,400	< LOQ	8,700	540	380	500	100	540
		3	130	25	< LOQ	240	2,400	< LOQ	6,300	620	220	450	94	480
		4	130	25	< LOQ	210	2,400	< LOQ	5,000	570	170	430	96	440
		6	130	26	< LOQ	190	2,300	< LOQ	3,000	560	88	380	97	350
		7	120	24	< LOQ	160	2,100	< LOQ	2,200	570	72	330	91	310

SI Table 23: Time series of measured biofilter effluent concentrations of relevant OMPs for the two sampling campaigns (SC) and the two biofilters (BF)

		Time	Metoprolol	Olmesartan	Primidone	Saccharin	Sitagliptin	SMX	Sucralose	Sulpiride	Telmisartan	Terbuthylazine	Terbutryn	Torasemide
		d	ng/L	ng/L	ng/L	ng/L	ng/L	ng/L	ng/L	ng/L	ng/L	ng/L	ng/L	ng/L
SC 1	BF 1	0.042	1,000	390	370	57	2,600	490	14,000	260	390	< LOQ	42	220
		0.25	680	400	340	35	2,500	530	14,000	270	460	< LOQ	51	220
		0.5	590	410	340	33	2,400	520	14,000	260	430	< LOQ	45	230
		1	380	420	370	14	2,300	550	15,000	260	410	< LOQ	44	230
		2	140	420	360	< LOQ	2,000	580	13,000	240	300	< LOQ	39	230
		3	53	390	330	< LOQ	1,700	540	12,000	230	190	< LOQ	33	200
		4	20	400	370	< LOQ	1,700	600	15,000	240	160	< LOQ	32	220
		6	2.4	400	350	< LOQ	1,300	430	14,000	210	130	< LOQ	30	200
		7	< LOQ	390	340	44	1,100	380	13,000	210	82	< LOQ	27	180
	BF 2	0.042	1,000	390	350	300	2,900	540	14,000	290	500	< LOQ	52	230
		0.25	850	380	360	320	2,800	510	14,000	280	550	< LOQ	55	220
		0.5	750	390	350	340	2,900	480	14,000	280	520	< LOQ	54	230
		1	540	400	350	120	2,700	520	14,000	290	490	< LOQ	51	240
		2	250	420	360	220	2,500	500	14,000	280	530	< LOQ	48	230
		3	110	400	370	25	2,400	470	14,000	280	450	< LOQ	43	230
		4	48	400	340	300	2,200	470	14,000	270	450	< LOQ	39	230
		6	12	420	360	< LOQ	1,800	350	14,000	270	410	< LOQ	36	230
		7	8.4	410	370	13	1,900	400	15,000	270	440	< LOQ	35	210
SC 2	BF 1	0.042	1,100	220	660	760	1,600	650	7,200	120	540	420	230	120
		0.25	1,100	230	710	840	1,700	700	6,700	120	560	510	300	120
		0.5	1,000	230	710	800	1,700	640	7,000	120	600	490	280	120
		1.25	820	210	700	730	1,600	590	7,000	120	550	480	280	120
		2	470	220	690	550	1,600	520	6,800	120	470	440	230	120
		3	280	220	730	250	1,600	490	6,300	120	410	430	230	120
		4	180	220	710	160	1,500	400	6,700	110	380	410	200	120
		6	88	220	710	71	1,300	320	6,600	110	280	380	180	110
		7	47	210	660	67	1,200	300	6,800	110	260	390	190	120
	BF 2	0.042	1,200	220	730	830	1,700	660	7,000	120	570	460	330	130
		0.25	1,100	230	730	850	1,700	640	6,500	120	560	490	360	120
		0.5	1,100	230	740	900	1,800	630	6,800	120	580	500	350	120
		1.25	730	230	730	820	1,800	560	6,900	130	610	520	350	130
		2	410	220	770	720	1,600	450	7,000	120	540	440	280	120
		3	200	220	710	480	1,500	340	6,800	120	570	450	250	120
		4	110	220	740	380	1,300	260	7,000	120	540	420	230	120
		6	39	220	710	110	1,200	200	7,300	120	520	420	220	120
		7	20	220	740	21	1,100	170	7,200	120	490	410	210	110

SI Table 24: Time series of measured biofilter effluent concentrations of relevant OMPs for the two sampling campaigns (SC) and the two biofilters (BF)

		Time	Tramadol	Trimethoprim	Valsartan	Venlafaxine	Xipamide
		d	ng/L	ng/L	ng/L	ng/L	ng/L
SC 1	BF 1	0.042	710	180	87	630	57
		0.25	620	170	68	560	57
		0.5	650	160	44	590	59
		1	610	150	15	530	56
		2	550	96	3.7	500	57
		3	500	59	< LOQ	450	55
		4	480	37	< LOQ	410	54
		6	430	14	< LOQ	360	59
		7	390	8.8	< LOQ	360	55
	BF 2	0.042	730	190	110	650	60
		0.25	610	180	97	540	56
		0.5	680	160	78	590	59
		1	680	140	47	580	59
		2	630	89	16	510	60
		3	580	59	6.1	500	59
		4	510	34	3.4	440	57
		6	500	10	< LOQ	390	56
		7	490	< LOQ	< LOQ	380	59
SC 2	BF 1	0.042	940	590	1400	930	15
		0.25	920	650	1400	900	14
		0.5	930	620	1200	940	13
		1.25	800	600	700	800	14
		2	790	500	190	770	14
		3	740	400	38	710	13
		4	710	320	14	700	13
		6	740	170	3.2	720	14
		7	600	120	2.2	580	13
	BF 2	0.042	990	600	1500	980	13
		0.25	920	610	1500	910	14
		0.5	970	600	1300	970	14
		1.25	780	560	880	750	15
		2	840	390	260	820	14
		3	750	230	58	710	13
		4	790	130	20	750	13
		6	830	38	6.7	790	13
		7	700	25	4.7	680	14

SI section 10: Model-based estimation of k_{biol} values

SI Table 25: Estimated k_{biol} values of all OMPs which had sufficient data available for model application. Available k_{biol} values from literature are also summarized. BF stands for biofilter and SC for sampling campaign.

OMP	Experimental						Literature values
	BF 1		BF 2				
	SC 1	SC 2	SC 1	SC 2	Mean value	Standard deviation	
	k_{biol} L/(g_{TSS}·d)						
Atenolol	1.084	1.003	1.157	1.208	1.113	± 0.077	< 0.13[1], < 1.5[2], 0.69 ± 0.05[3], 1.9 ± 0.2[7], 1.1 ± 0.1[8], 0.67 – 0.9[13], 0.53 – 0.67[14], 0.51 ± 0.03[15], 0.19 ± 0.05[16], 1.79 ± 0.18[17], 0.98 ± 0.04[18], 0.46 ± 0.06[19], 0.67 ± 0.03[20], 10.54 ± 1.05[21], 2.3 ± 0.03[22]
Azithromycin	0.136	0.148	0.238	0.436	0.240	± 0.120	< 0.13[1], < 1.5[2], 0.17 ± 0.16[11]
Bezafibrate	3.951	2.735	2.508	2.781	2.994	± 0.562	2.1 – 3.0[1], 3.4 – 4.5[2], 0.77 ± 0.34[10], > 2.9[11], 2.3 – 2.9[13], 5.2 – 6.0[14]
Caffeine	n.a.	0.401	n.a.	0.613	0.507	± 0.106	48.720 ± 4.440[4], 36.000 ± 3.528[5], 0.97[25], 4.05[26]
Carbendazim	0.006	0.064	0.032	0.089	0.048	± 0.031	< 0.1[8], 0.03 ± 0.01[17], 0.06 ± 0.01[18], 0.06[20], 0.12 ± 0.01[21], 0.11 ± 0.01[22]
Carbamazepine	0.000	0.000	0.000	0.000	0.000	± 0.000	0.168 ± 0.024[4], 0.240 ± 0.024[5], 0.154[9], < 0.008[19], < 0.005[11], < 0.1[13],[14], < 0.04[15],[16], 0 ± 0.01[17], -0.02 ± 0.03[18], 0[23], 0.007[24]
Citalopram	0.026	0.081	0.114	0.220	0.110	± 0.071	0.07 ± 0.09[15], 0.09 ± 0.09[16]
Clarithromycin	0.169	0.171	0.325	0.452	0.279	± 0.118	< 0.5[1], < 2[2], 0.2 ± 0.02[10], 0.034 ± 0.037[11], 0.18 – 0.29[13], < 0.2 [14], 0.25 ± 0.04[17], 0.33 ± 0.01[18], 0.26 ± 0.06[19], 0.11 ± 0.01[20], 4.22 ± 0.51[21], 1.15 ± 0.01[22]
Diatrizoate	0.045	0.035	0.017	0.092	0.047	± 0.028	< 0.1[1],[2], < 0.008[10], 0 ± 0.02[18]
Diclofenac	0.121	0.174	0.086	0.211	0.148	± 0.048	< 0.1[1],[2], 0.698 ± 0.048[4], 0.600 ± 0.048[5], 6.957[9], < 0.02 [11], < 0.1[13], 1.3 – 1.7[14], < 0.04[15], 0.10 ± 0.03[16], 0.05 ± 0.03[17], 7.39 ± 0.08[18], 0.81 ± 0.07[20], 0.06[21], 0.75 ± 0.01[22], > 0.77[23], 0.015[24]

OMP	Experimental						Literature values
	BF 1		BF 2				
	SC 1	SC 2	SC 1	SC 2	Mean value	Standard deviation	
	k_{biol}						
	L/(g_{TSS}·d)						
Fexofenadine	0.475	0.338	0.379	0.410	0.401	± 0.050	
Hydrochlorothiazide	0.040	0.142	0.047	0.104	0.083	± 0.042	< 0.2[13], < 0.1[14]
Ibuprofen	n.a.	27.375	n.a.	22.456	24.916	± 2.460	21 – 35[1], 9 – 22[2], 4.881[9], 1.33 ± 0.02[10], > 3[11], 29.24[23], 4.44[24]
Iomeprol	1.175	0.751	1.334	2.383	1.411	± 0.600	1.2 – 1.6[1], 0.5 – 1.0[2], 0.019 ± 0.009[10], 0.06 ± 0.013[11]
Iopamidol	0.033	0.206	0.050	0.252	0.135	± 0.095	< 0.36[1], < 0.1[2], < 0.01[10], 0.13 ± 0.01[20], 0.03 ± 0.01[21], 0.04 ± 0.01[22]
Iopromide	0.705	0.932	0.521	3.291	1.362	± 1.123	1.6 – 2.5[1], 1.0 – 2.0[2], 0.38 ± 0.05[17], 1.87 ± 0.43[18], 0.98 ± 0.39[19], 3.22 ± 0.1[20], 1.11 ± 0.02[21], 1.18 ± 0.03[22]
Irbesartan	0.056	0.143	0.121	0.196	0.129	± 0.050	
Metoprolol	1.615	1.738	2.535	2.746	2.159	± 0.490	0.58 ± 0.05[3], 0.35 ± 0.11[7], 0.69 – 0.76[13], 0.25 – 0.31[14], 0.15 ± 0.02[15], < 0.04[16], 0.44 ± 0.1[17], 0.3 ± 0.01[18], 0.16 ± 0.06[19], 0.27 ± 0.01[20], 3.22 ± 0.02[21], 4.58 ± 0.1[22], 0.128[25], 0,170[26]
Primidone	0.035	0.103	0.051	0.063	0.063	± 0.025	< 0.1[8], 0.022 ± 0.007[10], < 0.1[13],[14], 0.06 ± 0.01[17]
Sulfamethoxazole	0.009	0.459	0.091	1.108	0.417	± 0.436	7.368 ± 0.528[4], 5.880 ± 0.336[5], 0.12[6], 0.19 ± 0.03[10], 0.2 ± 0.02[11], 0.41[12], 1.68 ± 0.91[15], 0.72 ± 0.21[16], 0.28 ± 0.04[17], 0.29[18], 0.56 ± 0.05[19], 0.53 ± 0.05[20], 0.06 ± 0.02[21], -0.03 ± 0.01[22], 0.23[23], 0.07[24]
Telmisartan	0.024	0.027	0.000	0.000	0.013	± 0.013	
Tramadol	0.184	0.290	0.286	0.265	0.256	± 0.043	< 0.11[7], < 0.13[8], < 0.04[15],[16], 0.2 ± 0.01[17], 0.07 ± 0.01[22]
Trimethoprim	0.247	0.410	0.720	1.081	0.615	± 0.318	0.22 ± 0.022[11], < 0.3[13], 1.0 – 3.3[14], < 0.04[15], 0.08 ± 0.05[16], 0.55 ± 0.14[17], 11.45 ± 0.08[18], 0.13 ± 0.03[19], 0.51 ± 0.03[20], 1.83 ± 0.03[21], 3.04 ± 0.08[22], 0.04[23], 0.07[24]. 0.05[25]
Venlafaxine	0.140	0.267	0.288	0.278	0.243	± 0.060	0.15 – 0.2[13], < 0.1[14], < 0.04[15],[16], 0.05 ± 0.01[22]

(1) aerobic sludge from a CAS plant (SRT 11 ± 1 d), Joss et al. (2006)
(2) aerobic sludge from a MBR plant (SRT 30 – 40 d), Joss et al. (2006)
(3) aerobic sludge from a MBR pilot plant (SRT 20 d), assumption of 1 g_{TSS}/l ≈ 1 g_{COD}/l, Maurer et al. (2007)
(4) aerobic sludge from a CAS plant (SRT 6 d), Majewsky et al. (2011)

OMP	Experimental						Literature values
	BF 1		BF 2				
	SC 1	SC 2	SC 1	SC 2	Mean value	Standard deviation	
	k_{biol}						
	$L/(g_{TSS} \cdot d)$						

(5) aerobic sludge from a CAS plant (SRT 54 d), Majewsky et al. (2011)
(6) aerobic sludge from a CAS plant (SRT 7 d), Li and Zhang (2010)
(7) aerobic sludge from a CAS plant (STR 18 d), sludge sample taken in march, Wick et al. (2009)
(8) aerobic sludge from a CAS plant (STR 18 d), sludge sample taken in October, Wick et al. (2009)
(9) aerobic sludge from CAS plant, sludge samples stored in synthetic wastewater, low DOC concentrations during experiment, Urase and Kikuta (2005)
(10) aerobic sludge from a (single house) MBR, experiment 2 (SRT > 150 d), Abegglen et al. (2009)
(11) aerobic sludge from a (single house) MBR, experiment 2 (SRT > 100 d), Abegglen et al. (2009)
(12) aerobic sludge from a MLE activated sludge plant, Plósz et al. (2010)
(13) aerobic sludge from a hybrid CAS / MBBR plant (SRT 3 – 4 d), Falås et al. (2013)
(14) biofilm on carriers from a hybrid CAS / MBBR plant (oxic, same reactor as (13)), Falås et al. (2013)
(15) aerobic sludge from a CAS plant (SRT ≈ 30 d), Burzio et al. (2022)
(16) aerobic granular sludge (AGS) from a AGS plant (semi-continuous flow mode, SRT > 30 d), Burzio et al. (2022)
(17) aerobic sludge from a hybrid CAS / MBBR plant (SRT 3 d), Wolff et al. (2021)
(18) biofilm on carriers from a hybrid CAS / MBBR plant (oxic, same reactor as (17)), Wolff et al. (2021)
(19) aerobic sludge from a hybrid CAS / MBBR plant (SRT 5 d), Wolff et al. (2021)
(20) biofilm on carriers from a hybrid CAS / MBBR plant (oxic, same reactor as (19)), Wolff et al. (2021)
(21) aerobic sludge from a SBR hybrid activated sludge / MBBR plant (SRT 12 d), Wolff et al. (2021)
(22) biofilm on carriers from a hybrid CAS / MBBR plant (oxic, same reactor as (21)), Wolff et al. (2021)
(23) aerobic sludge from pilot-scale MBR, Fernandez-Fontaina et al. (2013)
(24) aerobic sludge from pilot-scale MBR used in a batch reactor setup, Fernandez-Fontaina et al. (2013)
(25) secondary sludge from an A^2/O-MBR plant (oxic tank), Xue et al. (2010)
(26) secondary sludge from an A^2/O-MBR plant (membrane tank), Xue et al. (2010)

SI Table 26: RMSE values and RMSE/S_0 ratios for model-based k_{biol} estimation. BF = biofilter and SC = sampling campaign

	BF1 SC1		BF1 SC2		BF2 SC1		BF2 SC2	
	RMSE	RMSE/ S_0	RMSE	RMSE/ S_0	RMSE	RMSE/ S_0	RMSE	RMSE/ S_0
	ng/L	-	ng/L	-	ng/L	-	ng/L	-
Atenolol	2.4	0.03	36.4	0.05	4.2	0.05	53.7	0.08
Azithromycin	10.9	0.06	58.4	0.07	13.0	0.07	77.3	0.09
Bezafibrate	1.6	0.03	80.9	0.10	2.3	0.05	103.6	0.13
Caffeine	n.a.(1)	n.a.(1)	28.6	0.05	n.a.(1)	n.a.(1)	38.1	0.06
Carbendazim	3.4	0.08	17.2	0.03	2.2	0.05	21.0	0.03
Carbamazepine	50.3	0.07	30.3	0.08	59.1	0.08	36.7	0.10
Citalopram	12.7	0.09	45.4	0.07	9.6	0.07	34.1	0.05
Clarithromycin	8.4	0.04	54.6	0.08	12.2	0.06	49.5	0.07
Diatrizoate	144.8	0.04	77.8	0.02	56.5	0.02	76.4	0.02
Diclofenac	112.9	0.05	63.7	0.05	126.0	0.05	107.4	0.08
Fexofenadine	4.6	0.03	16.7	0.03	5.1	0.03	18.3	0.03
Hydrochlorothiazide	247.4	0.06	61.2	0.02	239.4	0.06	137.1	0.05
Ibuprofen	n.a.(1)	n.a.(1)	15.8	0.03	n.a.(1)	n.a.(1)	3.1	0.01
Iomeprol	467.3	0.04	467.7	0.03	452.3	0.04	261.7	0.02
Iopamidol	7.4	0.11	56.0	0.08	12.0	0.17	48.5	0.07
Iopromide	19.8	0.12	60.6	0.07	17.2	0.11	26.4	0.03
Irbesartan	83.4	0.07	36.8	0.06	77.0	0.06	28.5	0.04
Metoprolol	41.6	0.03	52.8	0.04	14.7	0.01	75.6	0.05
Primidone	20.6	0.05	44.6	0.06	20.1	0.05	23.1	0.03
Sulfamethoxazole	65.5	0.12	20.9	0.03	32.3	0.06	20.3	0.03
Telsmisartan	111.2	0.29	119.9	0.24	175.1	0.46	136.6	0.28
Tramadol	49.3	0.06	44.0	0.04	49.6	0.06	62.4	0.06
Trimethoprim	10.1	0.05	45.0	0.06	8.5	0.04	60.2	0.08
Venlafaxine	35.1	0.05	46.8	0.05	37.6	0.05	69.4	0.07

(1) n.a.: no data available

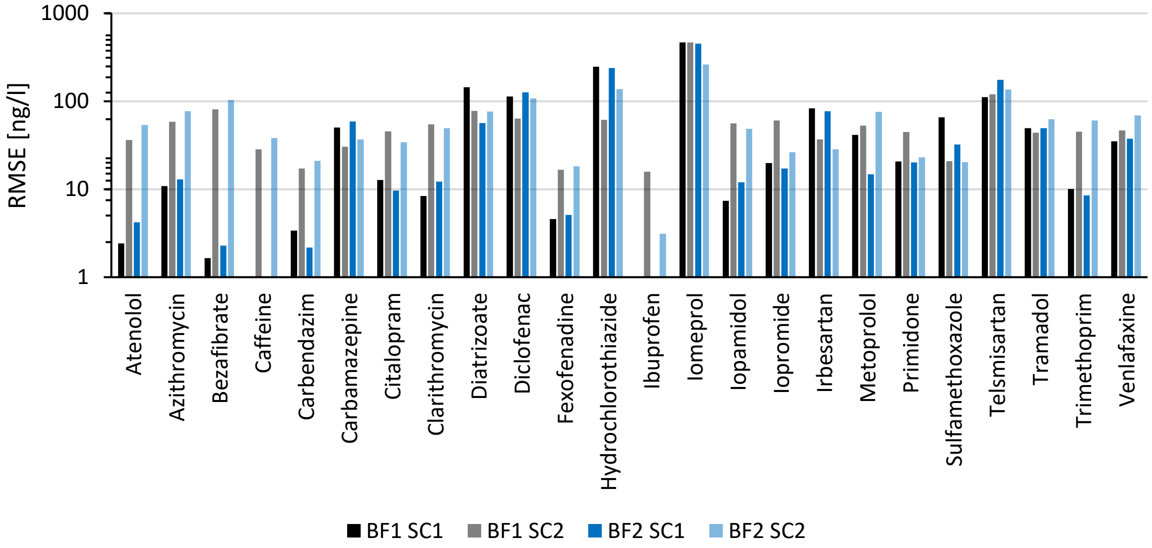

SI Figure 3: RMSE values for model-based k_{biol} estimation (BF = biofilter and SC = sampling campaign)

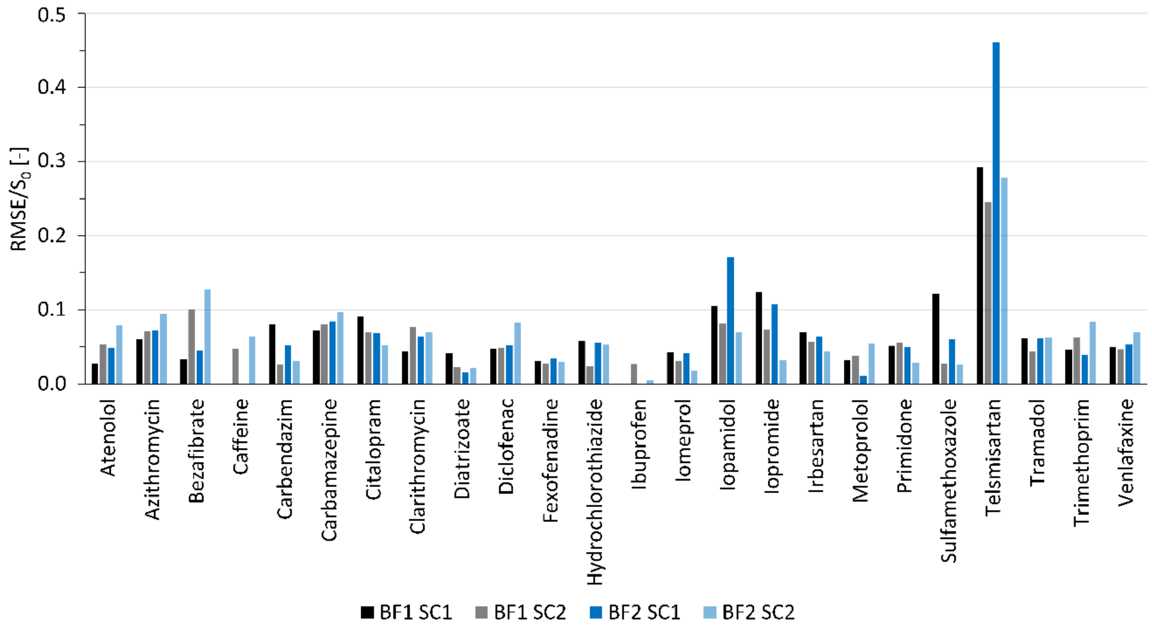

SI Figure 4: RMSE/S_0 ratios for model-based k_{biol} estimation (BF = biofilter and SC = sampling campaign)

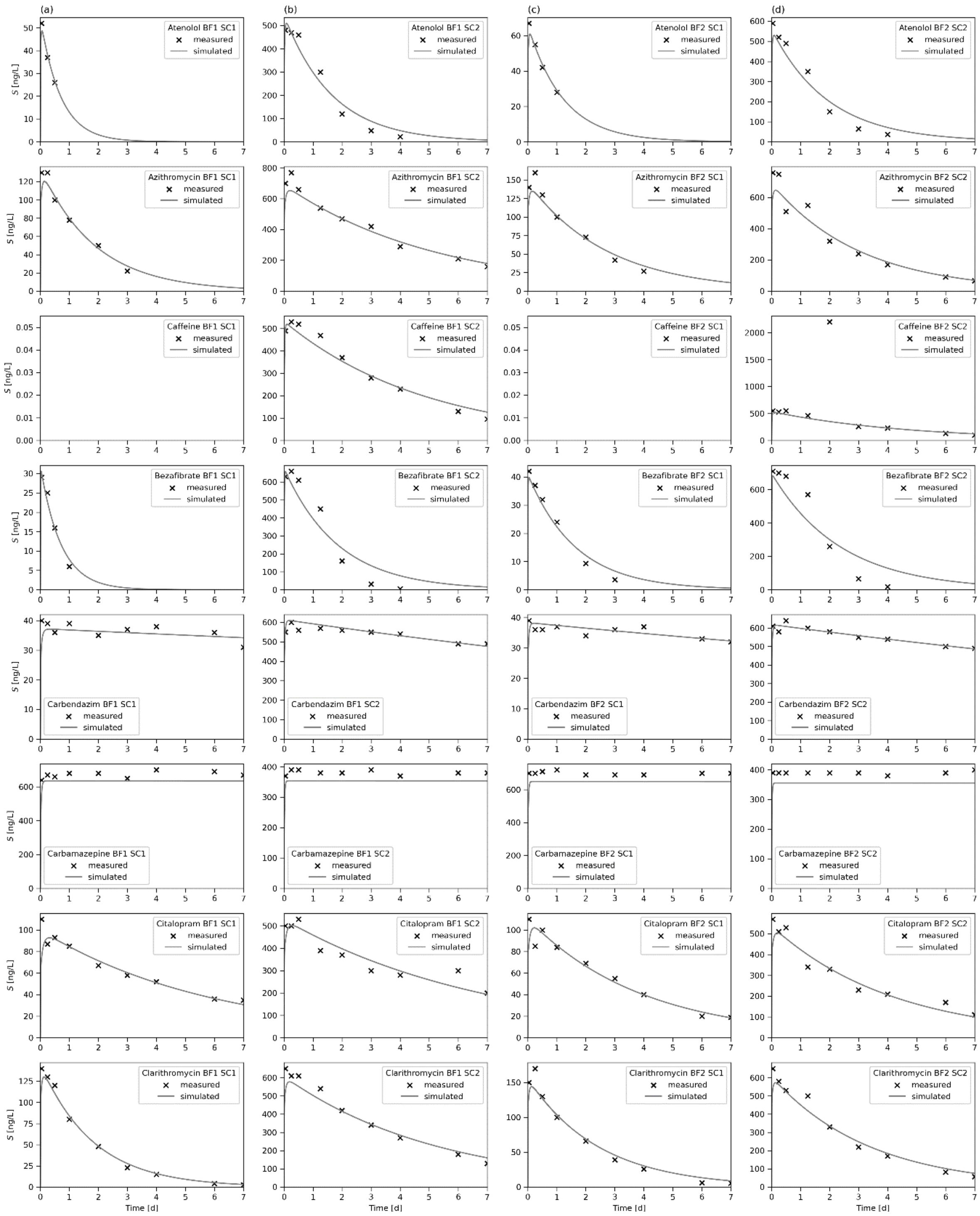

SI Figure 5: Graphical visualization of measured and simulated biofilter effluent concentrations plotted over the batch operation time. Columns of the grid represent the two biofilters (BF) and the two sampling campaigns (SC): (a) BF 1 SC 1, (b) BF 1 SC 2, (c) BF 2 SC 1 and (d) BF 2 SC 2. Datasets for OMPs atenolol, azithromycin, caffeine, bezafibrate, carbendazim, carbamazepine, citalopram and clarithromycin are displayed. In case of empty subplots, no experimental data was available (that met the defined quality criteria).

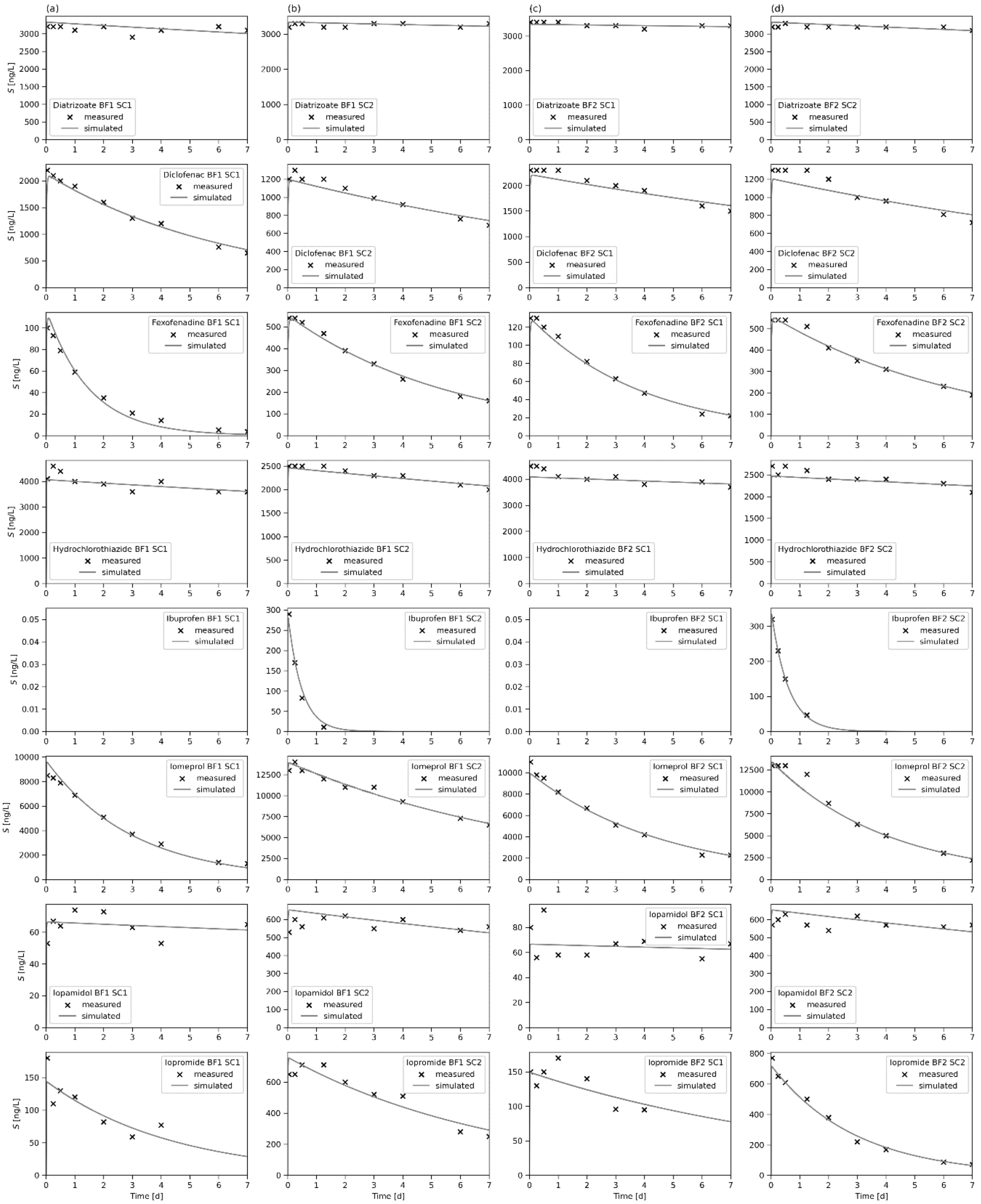

SI Figure 6: Graphical visualization of measured and simulated biofilter effluent concentrations plotted over the batch operation time. Columns of the grid represent the two biofilters (BF) and the two sampling campaigns (SC): (a) BF 1 SC 1, (b) BF 1 SC 2, (c) BF 2 SC 1 and (d) BF 2 SC 2. Datasets for OMPs diatrizoate, diclofenac, fexofenadine, hydrochlorothiazide, ibuprofen, iomeprol, iopamidol and iopromide are displayed. In case of empty subplots, no data was available (that met the defined quality criteria).

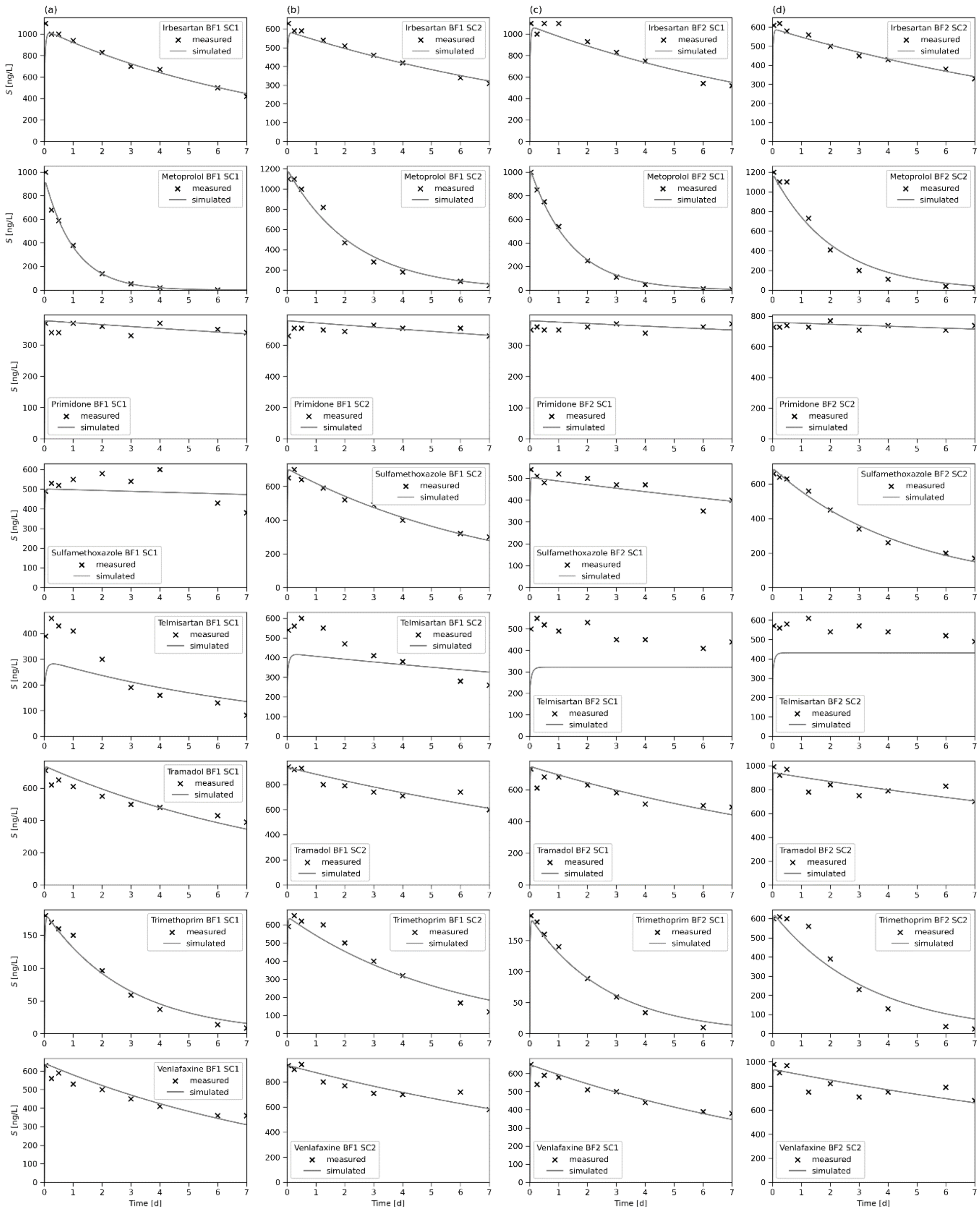

SI Figure 7: Graphical visualization of measured and simulated biofilter effluent concentrations plotted over the batch operation time. Columns of the grid represent the two biofilters (BF) and the two sampling campaigns (SC): (a) BF 1 SC 1, (b) BF 1 SC 2, (c) BF 2 SC 1 and (d) BF 2 SC 2. Datasets for OMPs irbesartan, metoprolol, primidone, sulfamethoxazole, telmisartan, tramadol, trimethoprim and venlafaxine are displayed. In case of empty subplots, no data was available (that met the defined quality criteria).

SI section 11: Outcome of the sensitivity analyses for the model describing the lab-scale biofilter setup

SI Figure 8 shows that the OMP concentration in the effluent of the biofilters relative to the initial concentrations at the start of the respective batch (expressed as S/S_0) was not sensitive to most of the model parameters that had to be assumed. The initial OMP concentration in the biofilter $S_{0,biofilter}$ was relevant mainly at the very start of the operation time. However, given that the resolution of the experimental data did not allow for fitting at this point in operation time, this model parameter was irrelevant for the estimation of k_{biol}. Consequently, $S_{0,biofilter}$ was assumed to be 0, independent of the considered OMP. The biofilm thickness L_{BF} was the only model parameter that had a significant influence on S/S_0 over the entire operation time, although its sensitivity function decreased once S/S_0 approached a value close to zero (see SI Figure 8 (a)). This behavior was in accordance with the definition of the absolute-relative sensitivity function as changes in model output become very small when the model output becomes small (Reichert, 1998). An increase in L_{BF} led to an increase in S/S_0, which is equivalent to a decrease in OMP removal efficiency. This was due to the dependence of the processes P1 – P3 (see Table 1) on the TSS concentration in the biofilm, which is ultimately also a function of L_{BF} (see SI Table 5 and SI Table 6). Uncertainty in the biofilm thickness was therefore the main driver of uncertainty in the predicted S/S_0 (see SI Figure 8 (e) – (h)).

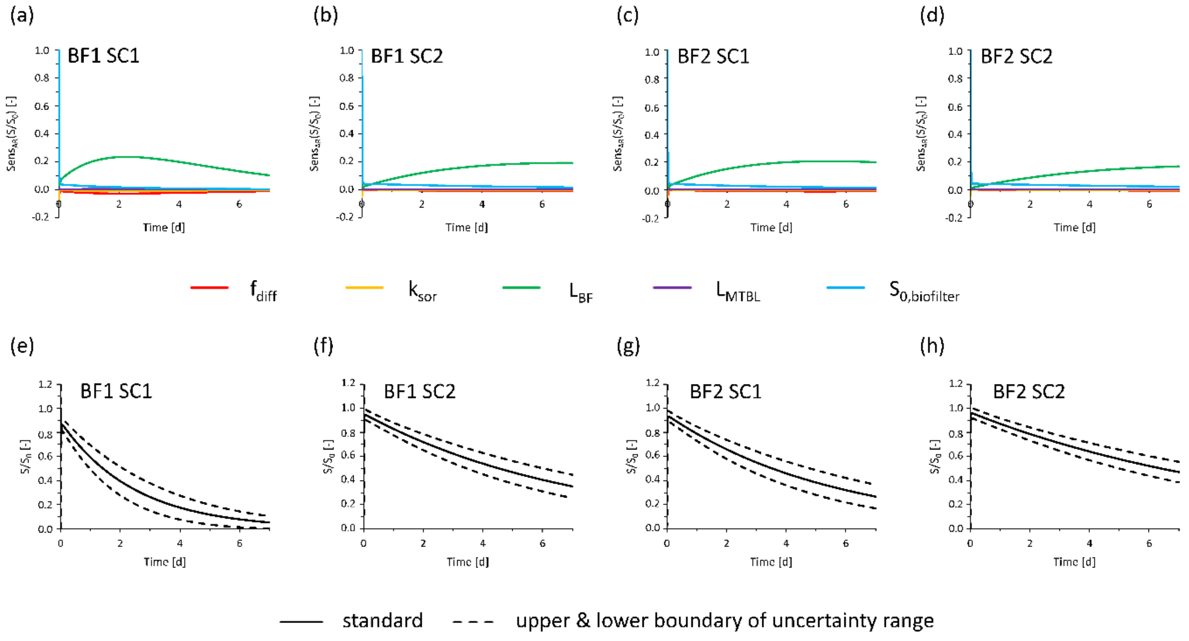

SI Figure 8: Absolute-relative sensitivity functions of the model parameters f_{diff}, k_{sor}, L_{BF}, L_{MTBL} and $S_{0,biofilter}$ regarding S/S_0 ((a) – (d)) and uncertainty ranges of S/S_0 resulting from uncertainty in these model parameters ((e) – (h)) for the four different TSS concentrations in the two biofilters (BF) during the two sampling campaigns (SC). S represents the biofilter effluent concentration and S_0 is the initial concentration of the batch.

Regarding the importance of individual transformation processes, SI Figure 9 shows that S/S_0 was insensitive regarding the dimensionless Henry's law constant H. Consequently, the exact value of H was irrelevant for the prediction of S/S_0, at least in the typical value range of most OMPs considered in this work (see SI Table 17). For relatively large values, H probably has a more pronounced effect on S/S_0 due to considerable volatilization in the aerated buffer tank. S/S_0 was very sensitive to both K_D and k_{biol}. The similar shape of the sensitivity functions can be

explained by the fact that biodegradation in the sorbed phase was considered in the model (see P2 in Table 1). For this reason, an increase in sorptive OMP removal (i.e. increasing K_D) is accompanied by an increase of OMP biodegradation in the sorbed phase. Eventually, this regenerates sorption capacity and leads to a constant sorptive OMP removal from the liquid phase. However, this results in a non-identifiability of k_{biol} and K_D. Thus, a simultaneous parameter estimation of the two parameters was not expedient.

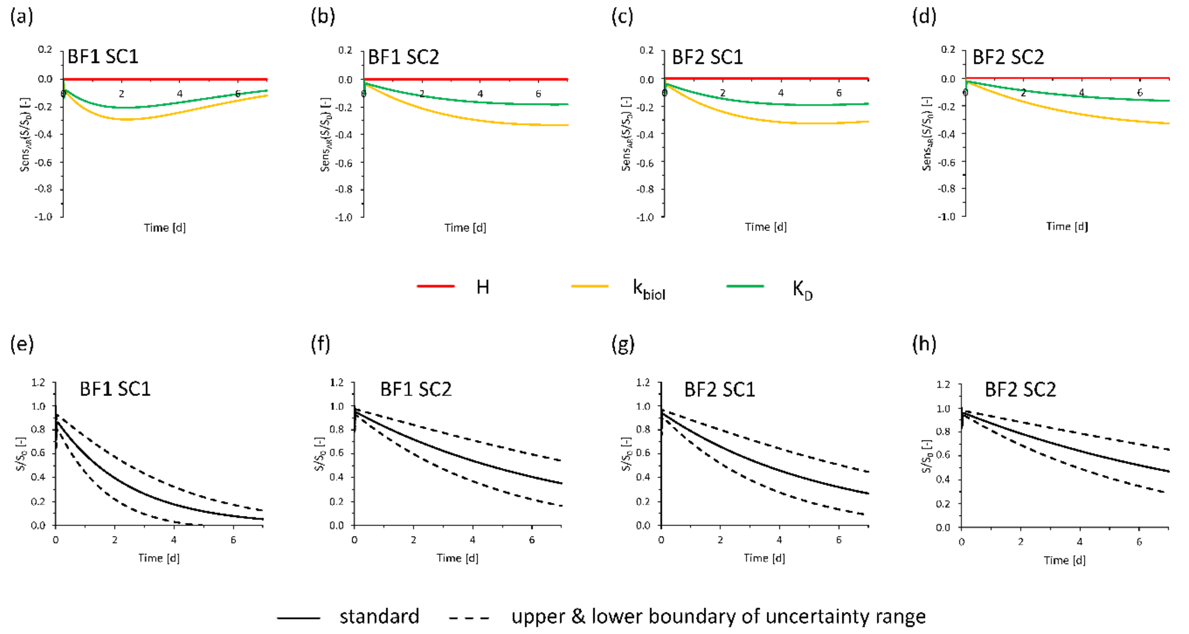

SI Figure 9: Absolute-relative sensitivity functions of the model parameters H, k_{biol} and K_D regarding S/S_0 ((a) – (d)) and uncertainty ranges of S/S_0 resulting from uncertainty in these model parameters ((e) – (h)) for the four different TSS concentrations in the two biofilters (BF) during the two sampling campaigns (SC). S represents the biofilter effluent concentration and S_0 is the initial concentration of the batch.

SI section 12: Outcome of the sensitivity analysis for the model used for conceptual evaluation of influencing factors on OMP biodegradation in biofilters for aWWT

In the modified model setup, effluent concentrations quickly reached a steady state since biomass growth and decay were not simulated and influent concentrations were kept constant. Consequently, an evaluation of the absolute-relative sensitivity function over the operation time as for the first model was not expedient. The absolute-relative sensitivity functions of the evaluated model parameters (for the steady state) are displayed in SI Figure 10 (a) and (b). As was expected from the previous sensitivity analysis for the model of the experimental setup, the biofilm thickness L_{BF} had a significant influence on the OMP effluent concentration with increasing L_{BF} leading to an increase in S/S_0 (defined as effluent concentration relative to the corresponding influent concentration S_0). The model parameter to which S/S_0 was most sensitive was the biomass concentration $VSS_{reactor}$. Increasing $VSS_{reactor}$ resulted in a decrease of S/S_0. Both parameters, L_{BF} and $VSS_{reactor}$, had a direct influence on the TSS concentration in the biofilm, which determines the rate expression of processes P1 – P3 and therefore the magnitude of both biodegradation and sorption in the biofilm (see Table 1, SI Table 5 and SI Table 6). To a certain degree, S/S_0 was also sensitive to f_{diff}. This highlights the importance of diffusive mass transfer for OMP removal during biofiltration. The effect of the other model parameters on S/S_0 was minor. In addition, S_0 did not influence S/S_0 at all. This can be traced back to the rate expressions of the processes P1 – P3 (see Table 1) being first order with regard to S. The sensitivity of S/S_0 regarding the selected model parameters increased with the value of k_{biol} and consequently with the biodegradability of the OMP under consideration. This was also reflected in the uncertainty ranges displayed in SI Figure 10 (c) and (d). K_D (i.e. the sorption affinity on biomass) had a similar, but less pronounced effect on the uncertainty of S/S_0 and its sensitivity to the selected model parameters (compare SI Figure 10 (a) and (b) as well as (c) and (d), respectively).

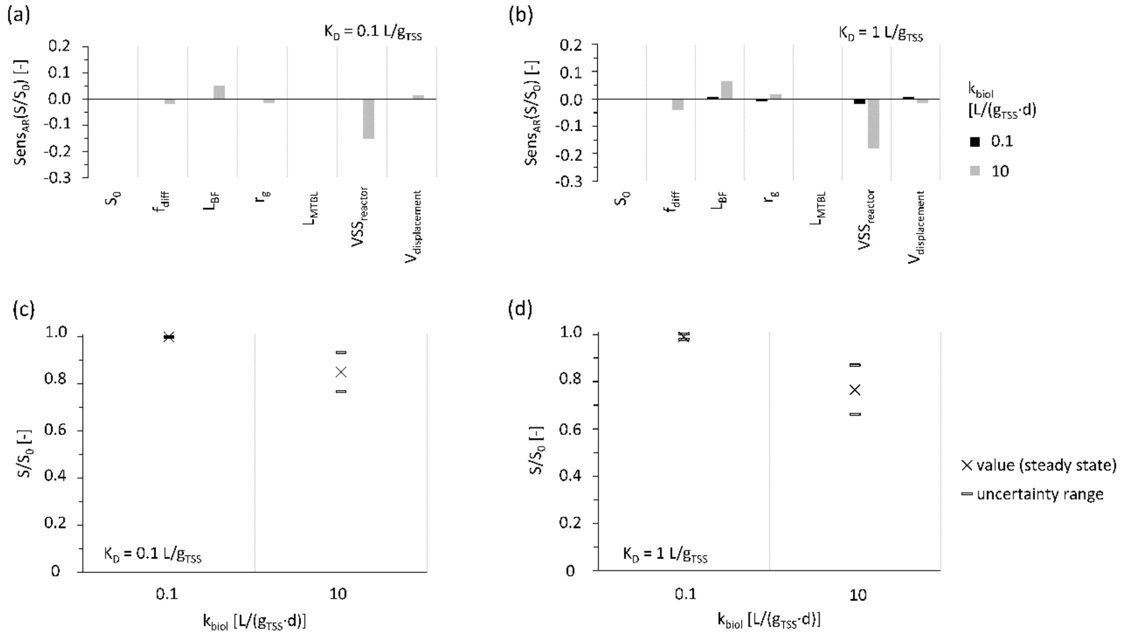

SI Figure 10: (a) and (b) absolute-relative sensitivity of relative OMP removal S/S_0 to selected model parameters for the second model setup at steady state for poorly biodegradable OMPs (k_{biol} 0.1 L/(g_{TSS}·d)) and well biodegradable OMPs (k_{biol} 10 L/(g_{TSS}·d)) and (c) and (d) corresponding uncertainty ranges for S/S_0. S represents the biofilter effluent OMP concentration and S_0 the corresponding influent concentration.

SI section 13: Comparison of predicted ranges of S/S_0 for individual OMPs with measured S/S_0 for sand filters used for aWWT as reported in literature

SI Figure 11 shows a comparison of the predicted ranges of S/S_0 for individual OMPs (based on Figure 4 (c)) with measured S/S_0 for sand filters operated under conditions typical for aWWT. The literature values are based on Reungoat et al. (2011), Zhang et al. (2017) and Zhiteneva et al. (2021). In all studies, sand filters were operated in parallel to GAC filters with identical feeds (secondary WWTP effluent in Reungoat et al. (2011) and Zhiteneva et al. (2021) and surface water in Zhang et al. (2017)) under similar operating conditions. Biomass concentrations were not reported in any of the studies. Reungoat et al. (2011) operated their sand filters with an EBCT of 120 min, Zhang et al. (2017) with 10 – 18 min and Zhiteneva et al. (2021) did not give the EBCT which was applied during the high resolution sampling campaign for which OMP removals were determined. In all three studies, OMP removals had to be determined visually from graphs. For Zhang et al. (2017), a mean value over all 7 runs was calculated.

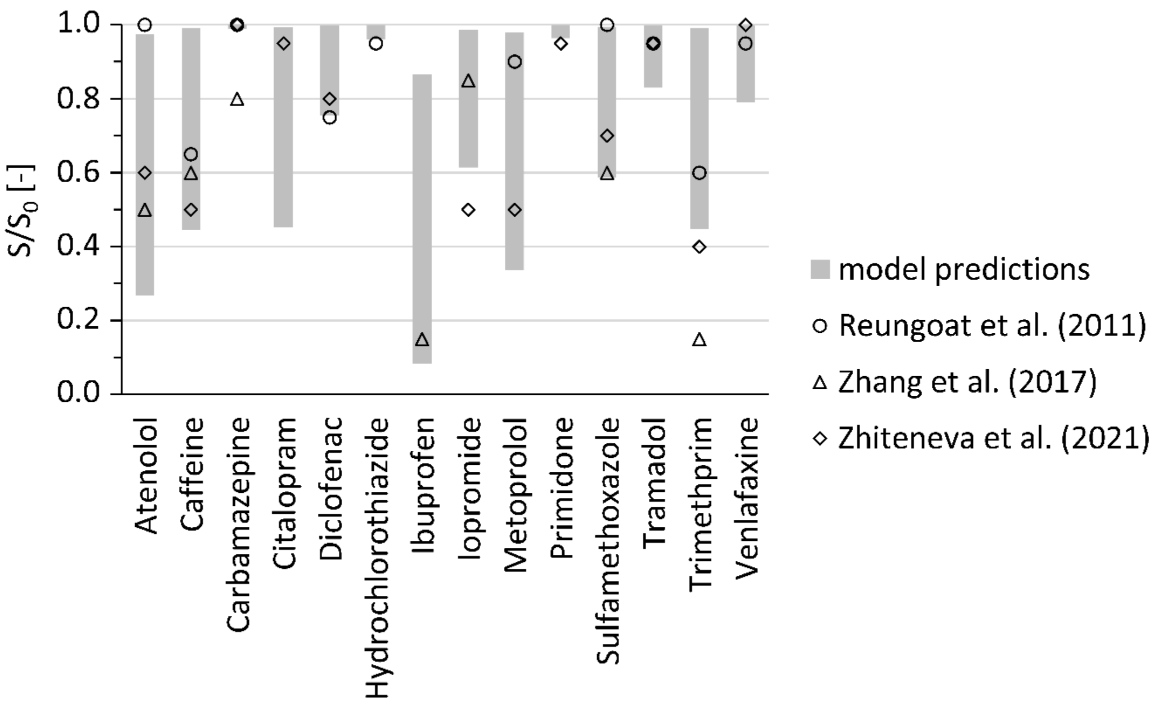

SI Figure 11: Measured relative OMP removal S/S_0 as reported in literature (Reungoat et al., 2011; Zhang et al., 2017; Zhiteneva et al., 2021) for sand filters that were operated in parallel to GAC filters in comparison to the corresponding S/S_0 ranges as predicted in this work (see Figure 4 (c))

SI section 14: List of acronyms or abbreviations used in the manuscript

SI Table 27: List of acronyms or abbreviations used in the manuscript

Acronym	Definition
aWWT	Advanced wastewater treatment
GAC	Granular activated carbon
LOQ	Limit of quantification
MTBL	Mass transfer boundary layer
OMP	Organic micropollutant
RMSE	Root mean square error
RSD	Relative standard deviation
TSS	Total suspended solids
WWTP	Wastewater treatment plant
VSS	Volatile suspended solids

SI nomenclature

Symbol	Name	Unit
$A_{biofilter}$	Cross-sectional area of the biofilter	L^2
A_F	External surface area of the filter bed material	L^2
B	OMP concentration sorbed on biomass	M/L^3
B_k	OMP concentration sorbed on biomass in biofilm layer k	M/L^3
$d_{biofilter}$	Inner diameter of the biofilter	L
D_L	Liquid-phase diffusion coefficient	L^2/T
EBCT	Emtpy bed contact time	T
f_{diff}	Molecular diffusivity reduction constant for diffusion in biofilms	-
H	Dimensionless Henry's law constant	-
$h_{biofilter}$	Biofilter bed height	L
k	Biofilm layer	-
k_{biol}	Pseudo first-order biodegradation kinetics constant	$L^3/(M \cdot T)$
K_D	Solid-water distribution coefficient	L^3/M
k_{sor}	Sorption rate constant	$L^3/(M \cdot T)$
L_{BF}	Total biofilm thickness	L
L_{MTBL}	Thickness of the mass transfer boundary zone	L
m_{TSS}	Absolute TSS mass in the reactor	M
MV_{OMP}	Molecular volume of OMP	L^3/N
MW_{OMP}	Molecular weight of OMP	M/N
n_{BF}	Number of biofilm layers	-
Q	Flow rate	L^3/T
q_G	Volume of air applied per volume of reactor and time	$L^3/(L^3 \cdot d)$
r_g	Mean grain radius of filter bed material	L
S_0	Initial concentration of OMPs in the buffer tank (recirculation setup) or OMP influent concentration (conceptual bGAC setup)	M/L^3
$S_{0,biofilter}$	Initial OMP concentration in the biofilter	M/L^3
S	OMP concentration	M/L^3

S_{BF1}	OMP concentration in first biofilm layer	M/L^3
$S_{biofilter,in}$	Influent OMP concentration into the biofilter	M/L^3
$S_{buffer\ tank}$	OMP concentration in the buffer tank	M/L^3
S_{bulk}	OMP concentration in the bulk volume of the biofilter	M/L^3
S_k	OMP concentration in biofilm layer k	M/L^3
S_{k+1}	OMP concentration in biofilm layer k+1	M/L^3
S_{k-1}	OMP concentration in biofilm layer k-1	M/L^3
t	Time	T
V_{BF}	Total volume of the biofilm	L^3
$V_{BF,i}$	Volume of one biofilm layer	L^3
$V_{biofilter}$	Biofilter volume	L^3
V_{buffer}	Volume of the buffer tank	L^3
V_{Bulk}	Bulk volume (biofilter)	L^3
$V_{displacement}$	Displacement volume of the filter bed material	L^3
X_{VSS}	Volatile suspended solids	M/L^3
$VSS_{reactor}$	VSS concentration in the biofilter with regard to the reactor volume	M/L^3
x	Spatial variable in direction of flow through the biofilter	L
X_{TSS}	Total suspended solids concentration	M/L^3
η	Viscosity of solution	$M/(L \cdot T)$
Θ_{bed}	Biofilter bed porosity	-
Θ_{BF}	Volume fraction of biofilm in $V_{biofilter}$	-
$\Theta_{BF,i}$	Volume fraction of one biofilm layer in $V_{biofilter}$	-
Θ_{Bulk}	Bulk volume fraction in $V_{biofilter}$	-
ρ_{OMP}	Mass density of OMP	M/L^3

References

Abegglen, C., Joss, A., McArdell, C., Fink, G., Schlüsener, M., Ternes, T., Siegrist, H., 2009. The fate of selected micropollutants in a single-house MBR. Water Research 43 (7), 2036–2046. doi:10.1016/j.watres.2009.02.005.

Burzio, C., Ekholm, J., Modin, O., Falås, P., Svahn, O., Persson, F., van Erp, T., Gustavsson, D., Wilén, B., 2022. Removal of organic micropollutants from municipal wastewater by aerobic granular sludge and conventional activated sludge. Journal of hazardous materials 438, 129528. doi:10.1016/j.jhazmat.2022.129528.

Çeçen, F., Aktas, Ö. (Eds.), 2011. Activated Carbon for Water and Wastewater Treatment. Wiley-VCH, Weinheim.

Chen, G., van Loosdrecht, M., Ekama, G., Brdjanovic, D., 2020. Biological Wastewater Treatment Principles, Modelling and Design: Principles, Modelling and Design. IWA Publishing, London.

Crittenden, J.C., Trussell, R.R., Hand, D.W., Howe, K.J., Tchobanoglous, G., 2012. MWH's water treatment: Principles and design, 3rd ed. ed. John Wiley and Sons, Hoboken, N.J.

Falås, P., Longrée, P., La Cour Jansen, J., Siegrist, H., Hollender, J., Joss, A., 2013. Micropollutant removal by attached and suspended growth in a hybrid biofilm-activated sludge process. Water Research 47 (13), 4498–4506. doi:10.1016/j.watres.2013.05.010.

Fernandez-Fontaina, E., Pinho, I., Carballa, M., Omil, F., Lema, J., 2013. Biodegradation kinetic constants and sorption coefficients of micropollutants in membrane bioreactors. Biodegradation 24 (2), 165–177. doi:10.1007/s10532-012-9568-3.

Fundneider, T., Acevedo, V., Wick, A., Albrecht, D., Lackner, S., 2021. Implications of biological activated carbon filters for micropollutant removal in wastewater treatment. Water Research 189. doi:10.1016/j.watres.2020.116588.

Göbel, A., Thomsen, A., McArdell, C., Joss, A., Giger, W., 2005. Occurrence and sorption behavior of sulfonamides, macrolides, and trimethoprim in activated sludge treatment. Environmental science & technology 39 (11), 3981–3989. doi:10.1021/es048550a.

Hayduk, W., Laudie, H., 1974. Prediction of diffusion coefficients for nonelectrolytes in dilute aqueous solutions. AIChE J. 20 (3), 611–615. doi:10.1002/aic.690200329.

Hermes, N., Jewell, K., Wick, A., Ternes, T., 2018. Quantification of more than 150 micropollutants including transformation products in aqueous samples by liquid chromatography-tandem mass spectrometry using scheduled multiple reaction monitoring. Journal of chromatography. A 1531, 64–73. doi:10.1016/j.chroma.2017.11.020.

Hörsing, M., Ledin, A., Grabic, R., Fick, J., Tysklind, M., La Cour Jansen, J., Andersen, H., 2011. Determination of sorption of seventy-five pharmaceuticals in sewage sludge. Water Research 45 (15), 4470–4482. doi:10.1016/j.watres.2011.05.033.

Hyland, K., Dickenson, E., Drewes, J., Higgins, C., 2012. Sorption of ionized and neutral emerging trace organic compounds onto activated sludge from different wastewater treatment configurations. Water Research 46 (6), 1958–1968. doi:10.1016/j.watres.2012.01.012.

Joss, A., Zabczynski, S., Göbel, A., Hoffmann, B., Löffler, D., McArdell, C., Ternes, T., Thomsen, A., Siegrist, H., 2006. Biological degradation of pharmaceuticals in municipal wastewater treatment: proposing a classification scheme. Water Research 40 (8), 1686–1696. doi:10.1016/j.watres.2006.02.014.

Kupper, T., Plagellat, C., Brändli, R.C., Alencastro, L.F. de, Grandjean, D., Tarradellas, J., 2006. Fate and removal of polycyclic musks, UV filters and biocides during wastewater treatment. Water Research 40 (14), 2603–2612. doi:10.1016/j.watres.2006.04.012.

Li, B., Zhang, T., 2010. Biodegradation and Adsorption of Antibiotics in the Activated Sludge Process. Environ. Sci. Technol. 44 (9), 3468–3473. doi:10.1021/es903490h.

Majewsky, M., Gallé, T., Yargeau, V., Fischer, K., 2011. Active heterotrophic biomass and sludge retention time (SRT) as determining factors for biodegradation kinetics of pharmaceuticals in activated sludge. Bioresource technology 102 (16), 7415–7421. doi:10.1016/j.biortech.2011.05.032.

Maurer, M., Escher, B., Richle, P., Schaffner, C., Alder, A., 2007. Elimination of beta-blockers in sewage treatment plants. Water Research 41 (7), 1614–1622. doi:10.1016/j.watres.2007.01.004.

Neef, J., Leverenz, D., Launay, M., 2022. Performance of Micropollutant Removal during Wet-Weather Conditions in Advanced Treatment Stages on a Full-Scale WWTP. Water 14 (20), 3281. doi:10.3390/w14203281.

Plósz, B., Leknes, H., Thomas, K., 2010. Impacts of competitive inhibition, parent compound formation and partitioning behavior on the removal of antibiotics in municipal wastewater treatment. Environmental science & technology 44 (2), 734–742. doi:10.1021/es902264w.

Radjenović, J., Petrović, M., Barceló, D., 2009. Fate and distribution of pharmaceuticals in wastewater and sewage sludge of the conventional activated sludge (CAS) and advanced membrane bioreactor (MBR) treatment. Water Research 43 (3), 831–841. doi:10.1016/j.watres.2008.11.043.

Reichert, P., 1998. AQUASIM 2.0 - User Manual, Dübendorf, Switzerland.

Reungoat, J., Escher, B.I., Macova, M., Keller, J., 2011. Biofiltration of wastewater treatment plant effluent: effective removal of pharmaceuticals and personal care products and reduction of toxicity. Water Research 45 (9), 2751–2762. doi:10.1016/j.watres.2011.02.013.

Rittmann, B.E., McCarty, P.L., 1980. Evaluation of steady-state-biofilm kinetics. Biotechnology and Bioengineering 22, 2359–2373.

Sathyamoorthy, S., Chandran, K., Ramsburg, C., 2013. Biodegradation and cometabolic modeling of selected beta blockers during ammonia oxidation. Environmental science & technology 47 (22), 12835–12843. doi:10.1021/es402878e.

Stevens-Garmon, J., Drewes, J., Khan, J., McDonald, J., Dickenson, E., 2011. Sorption of emerging trace organic compounds onto wastewater sludge solids. Water Research 45 (11), 3417–3426. doi:10.1016/j.watres.2011.03.056.

Takács, I., 2022. SUMO22. Dynamita, Sigale.

Ternes, T., Herrmann, N., Bonerz, M., Knacker, T., Siegrist, H., Joss, A., 2004. A rapid method to measure the solid-water distribution coefficient (Kd) for pharmaceuticals and musk fragrances in sewage sludge. Water Research 38 (19), 4075–4084. doi:10.1016/j.watres.2004.07.015.

Torresi, E., Polesel, F., Bester, K., Christensson, M., Smets, B., Trapp, S., Andersen, H., Plósz, B., 2017. Diffusion and sorption of organic micropollutants in biofilms with varying thicknesses. Water Research 123, 388–400. doi:10.1016/j.watres.2017.06.027.

Urase, T., Kikuta, T., 2005. Separate estimation of adsorption and degradation of pharmaceutical substances and estrogens in the activated sludge process. Water Research 39 (7), 1289–1300. doi:10.1016/j.watres.2005.01.015.

Wick, A., Fink, G., Joss, A., Siegrist, H., Ternes, T., 2009. Fate of beta blockers and psycho-active drugs in conventional wastewater treatment. Water Research 43 (4), 1060–1074. doi:10.1016/j.watres.2008.11.031.

Wolff, D., Helmholz, L., Castronovo, S., Ghattas, A., Ternes, T., Wick, A., 2021. Micropollutant transformation and taxonomic composition in hybrid MBBR - A comparison of carrier-attached biofilm and suspended sludge. Water Research 202. doi:10.1016/j.watres.2021.117441.

Worch, E., 2021. Adsorption Technology in Water Treatment: Fundamentals, Processes, and Modeling, 2nd ed. de Gruyter, Berlin/Boston.
Xue, W., Wu, C., Xiao, K., Huang, X., Zhou, H., Tsuno, H., Tanaka, H., 2010. Elimination and fate of selected micro-organic pollutants in a full-scale anaerobic/anoxic/aerobic process combined with membrane bioreactor for municipal wastewater reclamation. Water Research 44 (20), 5999–6010. doi:10.1016/j.watres.2010.07.052.
Zhang, S., Gitungo, S., Axe, L., Raczko, R., Dyksen, J., 2017. Biologically active filters - An advanced water treatment process for contaminants of emerging concern. Water Research 114, 31–41. doi:10.1016/j.watres.2017.02.014.
Zhiteneva, V., Drewes, J., Hübner, U., 2021. Removal of Trace Organic Chemicals during Long-Term Biofilter Operation. ACS EST Water 1 (2), 300–308. doi:10.1021/acsestwater.0c00072.
